# Photocatalysis‐Assisted Co_3_O_4_/g‐C_3_N_4_ p–n Junction All‐Solid‐State Supercapacitors: A Bridge between Energy Storage and Photocatalysis

**DOI:** 10.1002/advs.202001939

**Published:** 2020-10-01

**Authors:** Liqi Bai, Hongwei Huang, Songge Zhang, Lin Hao, Zhili Zhang, Hongfen Li, Li Sun, Lina Guo, Haitao Huang, Yihe Zhang

**Affiliations:** ^1^ Beijing Key Laboratory of Materials Utilization of Nonmetallic Minerals and Solid Wastes National Laboratory of Mineral Materials School of Materials Science and Technology China University of Geosciences Beijing 100083 P. R. China; ^2^ Key Laboratory of Synthetic and Biological Colloids Ministry of Education School of Chemical and Material Engineering Jiangnan University Wuxi 214122 P. R. China; ^3^ Department of Applied Physics The Hong Kong Polytechnic University Hung Hom Kowloon Hong Kong SAR 999077 P. R. China

**Keywords:** built‐in electric field, carbon nitride, energy storage materials, photocatalysis, p–n junction, supercapacitors

## Abstract

Supercapacitors with the advantages of high power density and fast discharging rate have full applications in energy storage. However, the low energy density restricts their development. Conventional methods for improving energy density are mainly confined to doping atoms and hybridizing with other active materials. Herein, a Co_3_O_4_/g‐C_3_N_4_ p–n junction with excellent capacity is developed and its application in an all‐solid‐state flexible device is demonstrated, whose capacity and energy density are considerably enhanced by simulated solar light irradiation. Under photoirradiation, the capacity is increased by 70.6% at the maximum current density of 26.6 mA cm^−2^ and a power density of 16.0 kW kg^−1^. The energy density is enhanced from 7.5 to 12.9 Wh kg^−1^ with photoirradiation. The maximum energy density reaches 16.4 Wh kg^−1^ at a power density of 6.4 kW kg^−1^. It is uncovered that the lattice distortion of Co_3_O_4_, reduces defects of g‐C_3_N_4_, and the facilitated photo‐generated charge separation by the Co_3_O_4_/g‐C_3_N_4_ p–n junction all make contributions to the promoted electrochemical storage performance. This work may provide a new strategy to enhance the energy density of supercapacitors and expand the application range of photocatalytic materials.

## Graphical abstract

Photoirradiation‐enhanced capacity behavior of Co_3_O_4_/g‐C_3_N_4_ p–n junction type all‐solid‐state supercapacitors is reported. The enhanced capacity and energy density under simulated solar light irradiation are ascribed to the built‐in electric field that facilitates the separation of photo‐generated charges. The structural distortion of Co_3_O_4_ under photoirradiation also accounts for the promoted electrochemical storage performance.

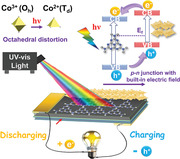

## 1 Introduction

Nowadays, the relying on fossil fuels has woken up human beings to concern about the sustainability of modern energy structure.^[^ ^1^ ^]^ Supercapacitors, with the advantages of high power density, fast discharging rate, long cycle life, and limited heat generation, have become a substitute for lithium ion batteries.^[^ ^2^ ^]^ For instance, Co_3_O_4_ as a typical transition metal oxide (TMO) exhibits excellent performance in supercapacitors and oxygen evolution reaction (OER),^[^ ^3^, ^4^ ^]^ because its octahedral centers in spinel‐structure are catalytically active, the charge is stored in Co^2+^/Co^3+^ state to produce reversible redox and it has a battery‐like faradaic behavior^[^ ^5^, ^6^, ^7^ ^]^ due to the formation of layered CoOOH intermediate with a large layer spacing as the case in Co(OH)_2_,^[^ ^8^ ^]^ which is beneficial to ion intercalation during the charge storage process. However, supercapacitors always have a low energy density, which is an enormous challenge for their applications.^[^ ^9^ ^]^

Photoirradiation‐mediation recently emerges as a promising strategy to promote the energy conversion and storage applications, such as hydrogen evolution reaction (HER),^[^ ^10^ ^]^ OER,^[^ ^11^ ^]^ oxygen reduction reaction,^[^ ^12^, ^13^, ^14^ ^]^ and rechargeable batteries.^[^ ^15^ ^]^ For instance, Wang et al. obtained an increased power density of Co_3_O_4_/Ni fibers/graphene electrodes after irradiation in the photo‐detecting applications.^[^ ^16^ ^]^ An et al. prepared Cu@Cu_2_O hybrid arrays and explored its photoirradiation‐enhanced capacity (PIEC) behavior in a three‐electrode system and attributed the PIEC to photo‐generated carriers.^[^ ^17^ ^]^ Chen et al. reported similar PIEC behavior of graphene/CNTs‐based electrode, in which the PIEC was ascribed to the photoconductive and photothermal effects of graphene.^[^ ^18^ ^]^

Graphitic carbon nitride (g‐C_3_N_4_) constructed by the tris–triazine units with a moderate bandgap of ∼2.7 eV is a promising low cost and nontoxic n‐type photocatalyst in artificial photosynthesis applications.^[^ ^19^, ^20^ ^]^ Recently, the potential of g‐C_3_N_4_ as a supercapacitor with fast electron transfer has been demonstrated.^[^ ^12^, ^21^, ^22^, ^23^, ^24^ ^]^ However, the electrical conductivity of g‐C_3_N_4_ is lower than that of carbon‐based materials, which limits its supercapacitor applications. Inspired by polarized photocatalytic materials,^[^ ^25^, ^26^ ^]^ constructing built‐in electric field for accelerated charge separation is a new strategy to enhance the energy storage capability of electrode materials,^[^ ^27^ ^]^ which has been mainly applied for promoting lithium, sodium, and aluminum ion storage,^[^ ^28^, ^29^, ^30^ ^]^ for example, Li et al. used the built‐in electric field of sulfurized Fe_2_O_3_ anode to reduce the activation energy and significantly improve the charge transfer kinetics in sodium batteries.^[^ ^31^ ^]^ Given the p‐type semiconductor feature of Co_3_O_4_, construction of p–n junction between Co_3_O_4_ and g‐C_3_N_4_ may be an ideal solution to enhance the energy density of supercapacitor devices. In particular, p–n junction‐based PIEC behavior and the fabrication of heterostructure‐based PIEC device has not been realized so far.

In this work, we fully utilized the advantages of light absorption, built‐in electric field and charge separation of Co_3_O_4_/g‐C_3_N_4_ (CoCN) p–n junction to realize PIEC behavior. Fascinatingly, we designed a flexible, all‐solid‐state, andsymmetricCoCN//CoCN supercapacitor device (ASSD) with PIEC function under photoirradiation. The capacity of the symmetrical supercapacitor is increased by 70.6% with photoirradiation, even at a high current density of 26 mA cm^−2^. The energy density is enhanced significantly from 7.5 to 12.9 Wh kg^−1^ at a power density of 16.0 kW kg^−1^ with photoirradiation, and its maximum energy density reaches 16.4 Wh kg^−1^ at a power density of 6.4 kW kg^−1^. In addition, the photocatalytic HER properties of CoCN heterostructure demonstrated the role of charge separation in promoting the energy density of supercapacitors, that is, photoirradiation promotes oxidation/reduction reactions via a built‐in electric field from the p–n junction.

## 2 Results and Discussion

### 2.1 Characterization of CoCN Heterojunction

**Figure 1:**
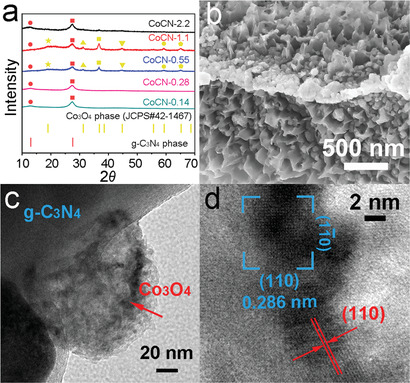
Characterization of the CoCN samples. a) XRD pattern of different samples. b) SEM, c) TEM and d) HRTEM images of the CoCN‐0.55 sample.

XRD patterns of a series of samples from CoCN‐0.14 to CoCN‐2.2 conform to the Co_3_O_4_ phase (JCPDS #42‐1467) (**Figure** **1**a). The peaks at 13.0° and 27.4° reveal the existence of g‐C_3_N_4_ phase, which correspond to the tri‐s‐triazine units of g‐C_3_N_4_ and its conjugated aromatic system, respectively.^[^ ^19^ ^]^ Cubic Co_3_O_4_ phase can be proved from the peaks located at 31.2°, 36.9°, 44.8°, 59.4°, and 65.2°, and they are noticeable in CoCN‐0.55 and CoCN‐1.1 samples. Interestingly, the peaks at 19° and 44.8° correspond to (111) and (100) planes of Co_3_O_4_, respectively, which are reported to be the planes abundant in active Co^2+^ species when they are exposed. Simultaneously, the (110) plane corresponding to 65.2° is related to Co^3+^ species,^[^ ^32^ ^]^ and thus CoCN‐0.55 and CoCN‐1.1 may be the best candidates for ultrafast surface oxidation/redox reactions with abundant exposed Co^3+^/Co^2+^ in an electrochemical supercapacitor. Scanning electron microscope (SEM) images of the CoCN‐0.55 sample reveal that Co_3_O_4_ nanospheres were embedded in the g‐C_3_N_4_ matrix (Figure 1b; Figure S2c and S5, Supporting Information), which is also confirmed by the transmission electron microscopy (TEM) (Figure 1c). This phenomenon could be due to the confinement effect of g‐C_3_N_4_, which limits the growth of Co_3_O_4_ and improves the dispersion of Co_3_O_4_ nanospheres. The interlayer distance of 0.286 nm corresponds to the (110) planes of Co_3_O_4_, which contain numerous electrochemically active Co^3+^ ions with an octahedral coordination.^[^ ^32^ ^]^ The (001) facet was exposed in CoCN‐0.55 by high resolution transmission electron microscope (HRTEM). For the spinel structure of Co_3_O_4_, the lattice fringes of 0.452 and 0.249 nm are attributed to (111) and (311) planes, respectively, which correspond to inactive Co^2+^ ions in an tetrahedral coordination.^[^ ^32^ ^]^ (Figure 1d; Figure S6, Supporting Information) Overall, the CoCN‐0.55 heterojunction has been constructed and may possess the potential in fast electrochemical energy storage applications.

### 2.2 Coordination and Surface States of CoCN Heterostructure Before and After Photoirradiation

**Figure 2:**
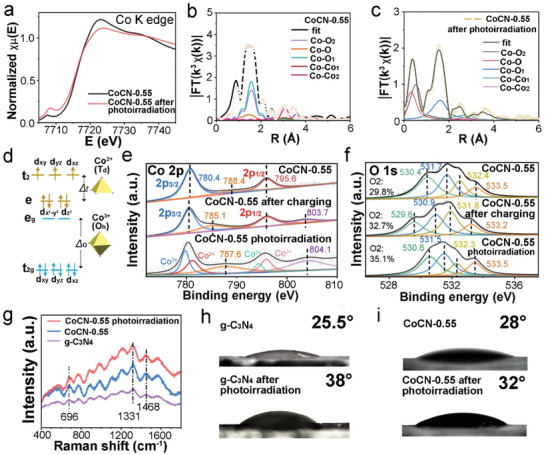
Coordination and surface states of CoCN heterostructure before and after photoirradiation. a) Co K‐edge XANES spectra of CoCN‐0.55 before and after photoirradiation. Fitted Fourier transforms *k* ^3^ *χ*(*k*) spectra b) before and c) after photoirradiation. d) Diagram of high‐spin tetrahedral coordination of Co^2+^ and low‐spin octahedral coordination of Co^3+^. XPS spectra of e) Co 2p and f) O 1s of CoCN‐0.55 before and after photoirradiation, and after charging. g) Raman spectra of g‐C_3_N_4_ and CoCN‐0.55 before and after photoirradiation. Contact angle measurement of h) g‐C_3_N_4_ and i) CoCN‐0.55 before and after photoirradiation.

X‐ray absorption fine structure spectroscopy (XAFS) measurement of Co K edge was carried out to reveal the coordination change of Co ions in CoCN before and after light irradiation. The distinctly different peaks in XANES represent the distortion of the [CoO_6_] octahedron, and the symmetry of the central inversion was changed due to the existence of carbon nitride and 3d energy splitting^[^ ^33^ ^]^ (**Figure** **2**a). The pre‐peak of Co K edge shifts to a higher energy state, indicating that the Co atom after photoirradiation is in a higher oxidation state.^[^ ^34^ ^]^ The increased pre‐peak intensity after photoirradiation is due to the change in the number of 3d electrons or the hybridization of 3d–2p orbitals,^[^ ^35^ ^]^ which is related to the geometric changes of TMO after photoirradiation.^[^ ^36^ ^]^ The oscillation amplitude of the K edge of CoCN‐0.55 after photoirradiation is significantly different from that before photoirradiation (Figure 2b,c; Figure S8–S9, Supporting Information), demonstrating that the local atomic arrangement and the coordination in [CoO_6_] octahedron were changed significantly. The octahedral structure of low‐spin Co^3+^ configuration and tetrahedral structure of high‐spin Co^2+^ configuration with their orbital energy levels are shown in Figure 2d. Interestingly, the EXAFS fitting data show that the Co—O_1_ and Co—O_2_ bonds become shorter after photoirradiation, which demonstrates the distortion of [CoO_6_] octahedrons with the stretching along the *c* axis^[^ ^33^, ^37^ ^]^ (Table S1, Supporting Information). Combined with the previous work in TMOs and layered Co(OH)_2_,^[^ ^8^, ^23^, ^38^, ^39^, ^40^ ^]^ the distortion of octahedral structure imparts a large single‐ion magnetic anisotropy and a low‐spin configuration of Co^3+^, which enhances the covalent component for the Co—O *σ* bond,^[^ ^38^, ^41^ ^]^ provides enough reactive active sites and exists in the form of CoOOH,^[^ ^39^ ^]^ which greatly improves the electrical conductivity and charge transfer efficiency of the materials, and is beneficial to the lowering of energy barrier of redox reactions on the surface of active materials.

X‐ray photoelectron spectroscopy (XPS) of CoCN‐0.55 before and after photoirradiation as well as before and after charging was conducted to further disclose the coordination evolution of Co ions (Figure 2e). The peaks could be fitted as Co 2p_3/2_ spin‐orbit peak (780.4 eV), satellite peak (788.4 eV), and Co 2p_1/2_ spin‐orbit peak (795.6 eV).^[^ ^42^ ^]^ After photoirradiation, the two satellite peaks at 787.6 eV and 804.1 eV becomes more obvious. They are related to the formation of oxygen vacancies due to the partial transformation of Co^3+^ to Co^2+^,^[^ ^5^, ^43^ ^]^ which is consistent with the results of oxygen vacancies under photoirradiation in previous studies.^[^ ^44^, ^45^ ^]^ It can be inferred that the Co_3_O_4_ phase has lattice distortions after photoirradiation, which agrees with the EXAFS fitted results and previous literature.^[^ ^46^ ^]^ After charging, the satellite peak at 788.4 eV shifts to 785.1 eV, revealing that Co_3_O_4_ was oxidized to CoOOH and CoO_2_ gradually.

O 1s spectra could help us understand the chemical variation of oxygen‐containing species after photoirradiation and charging (Figure 2f). The fitted peaks O1 (530.4 eV), O2 (531.7 eV), O3 (532.4 eV), and O4 (533.5 eV) located from low to high energy stand for Co—O, C=O, C—O, and C—OH/C—O—C in CoCN‐0.55, respectively.^[^ ^47^ ^]^ After photoirradiation, only lattice oxygen (O1) moved toward higher energy coupled with the decrease in Co—O electronic density, which is consistent with the Jahn–Teller distortion in [CoO_6_] octahedron.^[^ ^38^ ^]^ After charging, all of the peaks shift to lower energy, attributing to a partial reduction of Co^3+^ ions to Co^2+^ ions as reported in literature.^[^ ^48^ ^]^ Besides, the ratio of O_2_ increases after charging, revealing that more oxygen vacancies formed^[^ ^49^ ^]^ and the crystallinity of Co_3_O_4_ was destroyed partially. High‐resolution XPS N 1s spectra (Figure S10a,b, Supporting Information) can be fitted as pyridinic N (398.8 eV) and pyrrolic N (399.4 eV). Similarly, after charging, the peaks moved to higher energy because of the effect of high electronic‐supplied g‐C_3_N_4_ during redox reactions,^[^ ^50^, ^51^ ^]^ and the new peak at 406.0 eV confirms the formation of pyridine N‐oxide.^[^ ^52^, ^53^ ^]^

Raman spectra could help us reveal the molecular structure information of CoCN‐0.55 heterostructure after photoirradiation. The intensity of N—H bond peak (696 cm^−1^ )^[^ ^22^ ^]^ of CoCN‐0.55 heterostructure is higher than g‐C_3_N_4_ because of the bending hydrogen‐bond interaction (N—H^…^O—)^[^ ^54^ ^]^ outside the g‐C_3_N_4_ (001) planes. (Figure 2g) Interestingly, the peak area ratio of D band (1331 cm^−1^) and G band (1468 cm^−1^) (*S*_D_/*S*_G_) before and after photoirradiation is calculated to be 1.45 and 1.35, respectively, revealing that the surface defect and miscellaneous functional groups of g‐C_3_N_4_ are reduced, and the graphitization degree is increased after photoirradiation.^[^ ^55^, ^56^ ^]^ Therefore, it can be inferred that photoirradiation is beneficial to the improvement of the electrical conductivity of CoCN‐0.55 heterojunction. Besides, the contact angles of g‐C_3_N_4_ and CoCN‐0.55 increase after photoirradiation (Figure 2h,i), revealing the amphiphilic substance g‐C_3_N_4_ become more hydrophobic due to the reduced hydrophilic edge groups.^[^ ^57^ ^]^ The results mentioned above demonstrated that photoirradiation can affect the distortion of [CoO_6_] and reduce the surface miscellaneous functional groups of g‐C_3_N_4_ to promote conductivity. These findings provide an essential support to the explanation of the PIEC mechanism.

### 2.3 PIEC Behavior of CoCN‐0.55 Electrode in Three‐Electrode System

**Figure 3:**
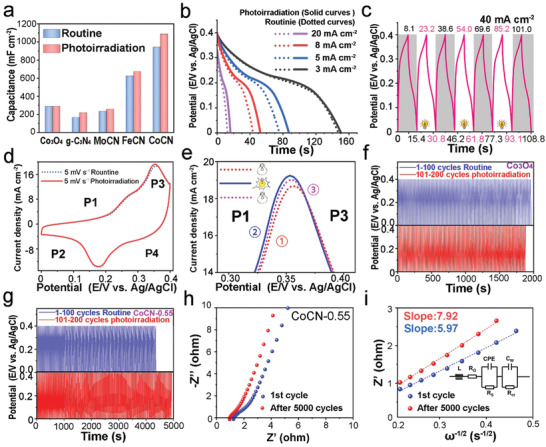
PIEC behavior of CoCN‐0.55 and other related electrodes in three‐electrode system. a) Capacity of Co_3_O_4_, g‐C_3_N_4_, MoCN, FeCN and CoCN‐0.55 electrodes with and without photoirradiation, b) GCD test of PIEC behavior and c) intermittent photoirradiation GCD tests of CoCN‐0.55 electrode with PIEC behavior, and its d,e) CV curves, f) PIEC behavior in CoCN‐0.55 electrodes GCD for 200 cycles, and g) no PIEC behavior exists in Co_3_O_4_ electrodes GCD for 200 cycles. h) EIS and i) fitted EIS results at low‐frequency region before and after 5000 GCD cycles.

To confirm the extraordinary electrochemical performance of CoCN‐0.55, g‐C_3_N_4_, MoO_3_/g‐C_3_N_4_ (MoCN), Fe_2_O_3_/g‐C_3_N_4_ (FeCN), and CoCN‐0.55 electrodes were prepared, and their galvanostatic charge–discharge (GCD) capacities with and without photoirradiation were compared. (**Figure** **3**a; Figure S12–S14 and Equation S1, Supporting Information) Interestingly, no apparent PIEC behavior happens on Co_3_O_4_ electrode, which is consistent with previous literature.^[^ ^16^ ^]^ With photoirradiation, the capacities of g‐C_3_N_4_, MoCN, FeCN, and CoCN‐0.55 electrodes are increased by 31.8%, 9.6%, 7.7%, and 15.3% at the same current density, respectively (Table S2, Supporting Information), demonstrating that g‐C_3_N_4_ semiconductor plays an important role in elevating capacity in g‐C_3_N_4_‐based heterojunctions due to the excellent photo‐generated carrier transport and separation ability of g‐C_3_N_4_.^[^ ^58^, ^59^ ^]^ CoCN‐0.55 also shows a high electrochemical capacity of 1519.5 mF cm^−2^ at a current density of 1.5 mA cm^−2^ in routine GCD measurement (Figure S15–S17 and Table S3–S4, Supporting Information), attributing to the octahedral spinel‐structure of Co_3_O_4_^[^ ^60^ ^]^ and the electronic supply effect of g‐C_3_N_4_. And the non‐linear GCD curves exhibits a battery‐like faradic activity^[^ ^7^ ^]^. To understand the PIEC behavior more deeply, routine, and photo‐irradiated GCD tests at different current densities were performed on CoCN‐0.55 electrode (Figure 3b and Table S5, Supporting Information). The capacity with photoirradiation increases by 2.0%, 15.4%, 20.5%, and 29.8% at the current densities of 3, 5, 8, and 20 mA cm^−2^, respectively. The enhancement in PIEC is more significant under a higher current density, and the charge and discharge time of GCD test under continuous photoirradiation increased (Table S6, Supporting Information), which confirms the effect of built‐in electric field. The intermittent photo‐irradiated GCD tests were executed at a constant current density of 40 mA cm^−2^ (Figure 3c). The discharge cycle time without and with photoirradiation was 7.3 and 7.6 s for the 1st off‐on cycle, 7.6 and 7.8 s for the 2nd off‐on cycle, 7.7 and 7.9 s for the 3rd off‐on cycle, respectively. Interestingly, the discharge time did not go back to the initial value when the photoirradiation was removed, which may be attributed to the distorted structure of Co_3_O_4_ after short‐term photoirradiation. Cyclic voltammetry (CV) measurements (Figure 3d) could help us understand the impacts of the built‐in electric field on electrochemical oxidation/reduction reactions. The CV profiles prove that CoCN‐0.55 sample exhibits a battery‐like faradaic charge storage behavior. P1/P2 peaks account for the reversible reaction between Co_3_O_4_ and CoOOH, and P3/P4 peaks stand for the reaction between CoOOH and CoO_2_.^[^ ^5^ ^]^ As shown in ① Routine GCD, ② Photoirradiation GCD, and ③ Routine GCD experiments (Figure 3e), CV curve in path ③ will not go back to path ①.

To further clarify the PIEC mechanism, two cyclic experiments were conducted separately on Co_3_O_4_ and CoCN‐0.55 electrodes. Even under photoirradiation, the GCD time of the Co_3_O_4_ electrode was reduced in the second 100 cycles (Figure 3f), due to the consumed Co_3_O_4_ species. This is in sharp contrast to the cyclic time of the CoCN‐0.55 electrode, which was increased visibly after 100 GCD cycles (Figure 3g), indicating that the PIEC behavior was derived from the photo‐generated carriers stored by g‐C_3_N_4_ rather than Co_3_O_4_ in the heterostructure under a continuous photoirradiation. Therefore, the constructed built‐in electric field formed by CoCN heterojunction promoted the oxidation/reduction reactions under photoirradiation.

Besides, electrochemical impedance spectroscopy (EIS) measurements were carried out to evaluate the cyclic performance of the system (Figure 3h; Figure S18, Supporting Information). The reduced EIS radius after 5000 GCD cycles indicates that the interface charge transfer resistance (*R*_ct_) of the CoCN‐0.55 electrode was reduced. More precisely, the Warburg coefficients (*ω* ^−1/2^) of the CoCN‐0.55 electrode were calculated to be 5.97 and 7.92 Ω s^−1/2^ before and after 5000 GCD cycles, respectively (Figure 3i; Equation S3, Supporting Information). Therefore, the initial diffusion coefficient of OH^−^ was calculated to be 2.16 × 10^−11^ cm^2^ s^−1^, and the diffusion coefficient after 5000 GCD cycles was 1.23 × 10^−11^ cm^2^ s^−1^ (Equation S4, Supporting Information). It discloses that the CoCN‐0.55 electrode still possesses a stable OH^−^ diffusion coefficient after cycling, where the large electrolyte contact area and low charge transport resistance are the main reasons (Notes in Figure S18, Supporting Information).

### 2.4 PIEC Behavior in CoCN//CoCN Supercapacitor Device Application

**Figure 4:**
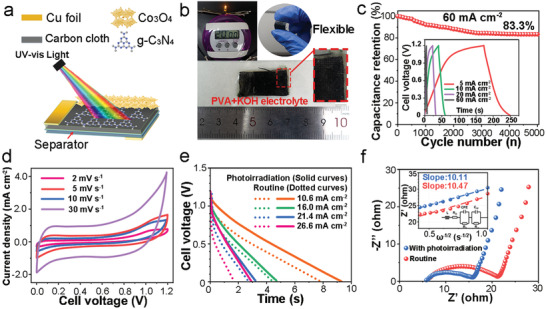
PIEC behavior in CoCN//CoCN supercapacitor devices. a) Concept and b) actual images of PIEC all‐solid‐state CoCN//CoCN flexible devices, c) Cyclic test up to 5000 cycles. Inset: GCD results, d) CV curves and e) PIEC behavior of the device, f) EIS and fitted ZRe−ω−1/2 curve of the device with and without photoirradiation.

Since the metal shell of the typical coin‐like supercapacitor device (Figure S19–S25, Supporting Information) or Swagelok‐type cell would hinder the light transmission to electrode, it is necessary to make a shell‐free supercapacitor device (CoCN//CoCN ASSD), which is composed of two carbon cloth electrodes, conductive copper foils, and a separator^[^ ^61^ ^]^ (**Figure** **4**a,b). The capacity is calculated to be 320.0, 165.0, 153.3, and 120.0 mF cm^−2^ at the current densities of 5, 10, 20, and 60 mA cm^−2^, respectively (Figure 4c inset). CV curves (Figure 4d) and the 83.3% capacity retention after 5000 GCD cycles (Figure 4c) reveal the significant battery‐like faradic activities and electrochemical cycle stability of as‐prepared ASSD, respectively. A LED was lighted up for more than 20 min with two devices connected in series (Figure 4b; Figure S29, Supporting Information). Under photoirradiation, the capacity of the device is calculated to be 82.2, 62.7, 57.1, and 64.3 mF cm^−2^ at the current densities of 10.6, 16.0, 21.4, and 26.6 mA cm^−2^, respectively, with the PIEC increment of 19.2%, 6.8%, 18.5%, and 70.6% (Figure 4e; Table S7, Supporting Information). Even if the solid‐state electrolyte may be evaporated during photoirradiation, the capacity increments are still more substantial than those in the three‐electrode system. Such achievement is attributed to the enhanced diffusion coefficient (Figure 4f), namely, with photoirradiation, the *R*_ct_ reduced, and the Warburg coefficient reduced from 10.47 to 10.11 Ω s^−1/2^ due to concentration polarization.^[^ ^62^ ^]^ Similarly, the diffusion coefficient of OH^−^ increases from 5.04 × 10^−12^ to 5.22 × 10^−12^ cm^2^ s^−1^.

The energy density reaches 12.9 Wh kg^−1^ even at a power density of 16.0 kW kg^‐1^ with photoirradiation, meanwhile, the energy density is only 7.5 Wh kg^−1^ at the same power density without photoirradiation (Table S8 and Equation S5–S6, Supporting Information). Besides, the maximum energy density is calculated as 16.4 Wh kg^−1^ at a power density of 6.4 kW kg^‐1^ under photoirradiation. Such an energy density could be obtained without sacrificing its power density.^[^ ^63^ ^]^ Ragone plot is essential to assess the overall performance of supercapacitor device by energy density (*E*) and power density (*P*) indexes^[^ ^64^ ^]^ (Figure S30, Supporting Information). Compared with the results in previous literatures, the as‐prepared device demonstrates an excellent *E–P* performance with photoirradiation, which is better than some carbon‐based asymmetric supercapacitor devices, proving that photoirradiation is an efficient approach to enhance energy density of supercapacitors.

### 2.5 Photocatalytic and Photoelectrochemical Performance of CoCN Photocatalysts

**Figure 5:**
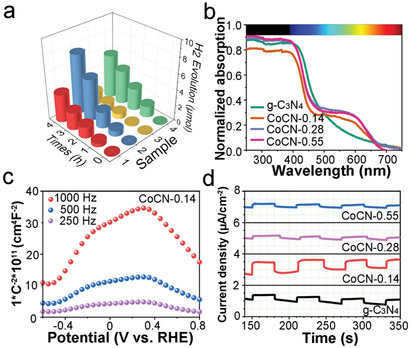
Photocatalytic activity and photoelectrochemical characterization of g‐C_3_N_4_ and CoCN photocatalysts. a) Photocatalytic H_2_ production amounts (sample 1–4 is g‐C_3_N_4_, CoCN‐0.14, CoCN‐0.28, and CoCN‐0.55, respectively). b) DRS and c) Mott–Schottky curves of CoCN‐0.14 at different frequencies. d) Photocurrent responses.

Photocatalytic HER experiments without co‐catalyst Pt were carried out to verify the mechanism of the role of CoCN heterojunction in PIEC performance (**Figure** **5**a). CoCN‐0.14 shows the highest H_2_ production yield of 38.0 µmol g^−1^ h^−1^ within 4h, which is 2.1 times the amount of pure g‐C_3_N_4_ (Table S9, Supporting Information), indicating the much higher photocatalytic activity of CoCN‐0.14. This may be attributed to the enhanced charge separation in the Co_3_O_4_/g‐C_3_N_4_ heterojunction.^[^ ^20^, ^65^ ^]^ UV–vis diffuse‐reflectance spectra (DRS) show that the absorption edge of g‐C_3_N_4_ is close to 700 nm with a bandgap of 2.06 eV based on Kubelka–Munk function,^[^ ^66^ ^]^ (Figure S31, Supporting Information), which is consistent with the characteristics of nitrogen vacancies in g‐C_3_N_4_.^[^ ^67^, ^68^ ^]^ With the increment of Co_3_O_4_ content, the photoabsorption of CoCN increases (Figure 5b). The p‐type space charge region and the n‐type space charge region are shown in Mott–Schottky curves (Figure 5c), indicating the formation of the p–n junction in CoCN (Note in Figure 5c). The transient photocurrent measurements reveal that CoCN‐0.14 shows the most vigorous anodic current response among all the photoelectrodes, indicating that the fabrication of p–n junction even with a small amount of Co_3_O_4_ can effective hinder the recombination of photogenerated electrons and holes during the photocatalytic process.^[^ ^68^, ^69^ ^]^ (Figure 5d; Figure S33–S36, Supporting Information)

### 2.6 Mechanism of PIEC Behavior in CoCN p–n Junction Supercapacitors

**Figure 6:**
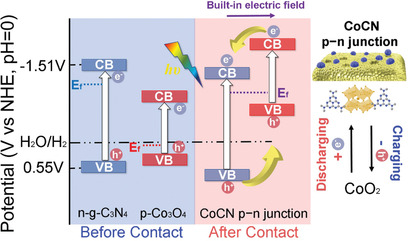
The mechanism of the PIEC behavior in CoCN p–n junction supercapacitor.

The band structure of n‐type g‐C_3_N_4_ and p‐type Co_3_O_4_ are shown in **Figure** **6**. Before contact, the conduction band (CB) of Co_3_O_4_ was lower than that of g‐C_3_N_4_. After the construction of p–n junction, the energy levels of g‐C_3_N_4_ shift down, meanwhile, those of Co_3_O_4_ shift up till the Fermi level (*E*_f_) equilibrium between g‐C_3_N_4_ and Co_3_O_4_ was achieved. Finally, the CB bottom and valence band (VB) top of Co_3_O_4_ were higher than those of g‐C_3_N_4_. Under photoirradiation, the electron–hole pairs were generated from g‐C_3_N_4_ (Equation 5). Driven by the potential gradient of the p–n junction, the electrons are transferred from the CB of Co_3_O_4_ to that of g‐C_3_N_4_. Meanwhile, the photoinduced holes are more prone to be accumulated to the VB of Co_3_O_4_.^[^ ^70^, ^71^ ^]^ Thus, efficient charge separation occurs, rendering longer surviving electrons and holes. The total oxidation reactions of charging processes (Equation 6) and total reduction reactions of discharging processes of CoCN p–n junction (Equation 7) are thereby promoted.

As reported in literature,^[^ ^8^ ^]^ the surface oxidation reactions of Co_3_O_4_ without photoirradiation during charge process are:^[^ ^5^ ^]^

(1) $$\begin{array}{c} Co_{3}O_{4}+OH^{-}+H_{2}O\to 3\mathrm{CoOOH}+e^{-} \end{array}$$

(2) $$\begin{array}{c} \mathrm{CoOOH}+OH^{-}\to \mathrm{Co}O_{2}+H_{2}O+e^{-} \end{array}$$

and the reduction reactions without photoirradiation during discharge processes are:^[^ ^42^ ^]^

(3) $$\begin{array}{c} \mathrm{Co}O_{2}+H_{2}O+e^{-}\to \mathrm{CoOOH}+OH^{-} \end{array}$$

(4) $$\begin{array}{c} 3\mathrm{CoOOH}+e^{-}\to Co_{3}O_{4}+OH^{-}+H_{2}O \end{array}$$

with photoirradiation, due to^[^ ^72^ ^]^

(5) $$\begin{array}{c} g-C_{3}N_{4}+nh\nu \to nh^{+}+ne^{-} \end{array}$$

g‐C_3_N_4_ with abundant N species possesses chemical anchoring centers, high electron density, and electron supply effect,^[^ ^22^, ^55^ ^]^ the photocatalysis‐assisted charging process of the heterojunction may be described as,

(6) $$\begin{array}{ccc} & & g-C_{3}N_{4}+{\mathrm{Co}}_{3}O_{4}+4{\mathrm{OH}}^{-}+n\mathrm{hv}\to g-C_{3}N_{4}+3\mathrm{Co}O_{2}+2H_{2}O\\ & & \;+\;\left(n+4\right)e^{-}+nh^{+} \end{array}$$

and the photocatalysis‐assisted discharging processes may be:

(7) $$\begin{array}{ccc} & & g-C_{3}N_{4}+3\mathrm{Co}O_{2}+2H_{2}O+n\mathrm{hv}\to g-C_{3}N_{4}+{\mathrm{Co}}_{3}O_{4}+4{\mathrm{OH}}^{-}\\ & & \;+\;\left(n-4\right)e^{-}+nh^{+} \end{array}$$

where h^+^ stands for photogenerated holes and e^−^ represents photogenerated electrons or free electrons in oxidation/reduction reactions, *n* is the number of photogenerated carriers. As the number of photogenerated carriers increases from 0 to *n* with photoirradiation, more electrons are stored as charges and separated from the holes by the built‐in electric field formed by p–n junction. (Equation (6), (7)). Thus, the GCD capacity increases (Equation S7, Supporting Information). Accordingly, the energy density (*E*) also enhances (Equation S8, Supporting Information).

## 3 Conclusions

In summary, the PIEC was surveyed based on the Co_3_O_4_/g‐C_3_N_4_ (CoCN) p–n junction electrode and its all‐solid‐state flexible device. With photoirradiation, the capacity was increased by 29.8% even at a high current density of 20 mA cm^−2^ in a three‐electrode system. The GCD capacity of the CoCN//CoCN ASSD device rises by 70.6% at a high current density of 26.6 mA cm^−2^. The energy density was enhanced from 7.5 to 12.9 Wh kg^−1^ even at a high power density of 16.0 kW kg^‐1^ with photoirradiation. The considerably enhanced electrochemical performance originates from the [CoO_6_] lattice distortion, increased interfacial hydrophobicity and promoted photo‐generated charges separation of Co_3_O_4_/g‐C_3_N_4_ p–n junction. This study discloses the tremendous potential of p–n junction‐based electrode for high energy density supercapacitor applications and may inspire further development of other photoirradiation‐enhanced electrochemical devices for energy conversion and storage.

## 4 Experimental Section

This section is available in the Supporting Information.

## Conflict of Interest

The authors declare no conflict of interest.

## Author contributions

L.Q.B. and H.W.H. gave the original idea of the work. L.Q.B. designed the experiments, made the data processing and wrote the paper, H.W.H. guided this project and revised this manuscript. S.G.Z. provided valuable aid in partial material preparation, and L.H. provided a crucial assist in drawing figures of the paper. Z.L.Z. and L.N.G. helped in sample preparation and characterization during the manuscript revision. H.F.L. contributed to the photocatalytic HER test. L.S. contributed to the data analysis. H.T.H. guided this work, revised this manuscript and made manuscript preparation. Y.H.Z. guided this project and provided funds. All co‐authors commented on the manuscript.

## Supporting information

Supporting Information
