# Supplementary material for: Photocatalysis‐Assisted Co3O4/g‐C3N4 p–n Junction All‐Solid‐State Supercapacitors: A Bridge between Energy Storage and Photocatalysis
Source: Adv Sci (Weinh). 2020 Oct 1;7(22):2001939. doi: 10.1002/advs.202001939 (PMC7675041; doi:10.1002/advs.202001939)
Supplement: Supplementary file 1 — Supporting Information [file ADVS-7-2001939-s001.pdf]

© 2020 Wiley-VCH GmbH

ADVANCED SCIENCE
Open Access

# Supporting Information

for *Adv. Sci.,* DOI: 10.1002/advs.202001939

Photocatalysis-Assisted $Co_3O_4$/g-$C_3N_4$ p–n Junction All-Solid-State Supercapacitors: A Bridge between Energy Storage and Photocatalysis

*Liqi Bai, Hongwei Huang,* Songge Zhang, Lin Hao, Zhili Zhang, Hongfen Li, Li Sun, Lina Guo, Haitao Huang,* and Yihe Zhang**

# Supporting Information

## Photocatalysis-assisted $Co_3O_4$/g-$C_3N_4$ p-n junction all-solid-state supercapacitors: A bridge between energy storage and photocatalysis

*Liqi Bai, Hongwei Huang *, Songge Zhang, Lin Hao, Zhili Zhang, Hongfen Li, Li Sun, Lina Guo, Haitao Huang *, and Yihe Zhang **

Correspondence and requests for materials should be addressed to Y.Z (email: zyh@cugb.edu.cn) or H.H. (email: aphhuang@polyu.edu.hk) or H.H. (email: hhw@cugb.edu.cn).

# Content

1. Experimental Section .......................................................... 3

2. Equations in Text .......................................................... 7

3. Supplemental Figures and Tables ........................................ 8

4. References .......................................................... 45

# 1. Experimental Section

**Synthesis of g-$C_3N_4$.**

Typically, 5 g melamine was calcined in an alumina crucible at argon atmosphere at 520 °C for 3 h with a heating rate of 5 °C/min. Then 0.10 g g-$C_3N_4$ was dissolved in 20 ml deionised water with ultrasonic stirring for 30 min. The acquired solution was recorded as CN-20. After hydrothermal reaction at 160°C for 3h, the precipitates were separated by centrifugation and washed with deionised water and alcohol for several times. In the end, g-$C_3N_4$ was obtained after drying in vacuum at 60 °C for overnight.

**Preparation of clean Ni foam and carbon cloth**

1×1 $cm^2$ Ni foam with a thickness of 1.5 mm was soaked in dilute hydrochloric acid for more than 12 h, the clean nickel foam was obtained after washing with alcohol and deionised water for several times.

Carbon cloth (Carbon energy technology co. Ltd., Taiwan, China) was immersed in a mixed solution (concentrated sulfuric acid and concentrated nitric acid with the volume ratio about 1:1) for 24h.[1] Then the treated carbon cloth was washed with alcohol and deionised water for several times.

**Synthesis of CoCN-0.55 electrodes and other related electrodes**

Typically, 0.55 mmol $Co(NO_3)_2{\cdot}6H_2O$ was dissolved in 20 ml deionised water with moderate stirring for 30 min. Then, the as-obtained solution, CN-20 and clean Ni foam were transferred to a 50ml Teflon-lined stainless autoclave for hydrothermal reaction at 140 °C for 9 h[2]. After that, the electrodes were taken out and dried in a vacuum oven at 60 °C for overnight. The CoCN-0.55 electrodes were obtained after annealing the as-prepared Ni foams at 400 °C with a heating rate of 3 °C/min for 3 h. CoCN-0.55 sample was prepared in a similar manner without adding Ni foam. The pure $Co_3O_4$ electrode was prepared without adding CN-20 solution. The mass loading of CoCN-0.55 on Ni foam and carbon cloth is ~1.0 and ~2.2 mg $cm^{-2}$, respectively.

Similarly, CoCN-0.14, CoCN-0.28 and CoCN-1.1 samples were prepared separately by dissolving 0.14, 0.28, and 1.1 mmol $Co(NO_3)_2{\cdot}6H_2O$ in the same procedure.

**Synthesis of FeCN electrode and MoCN electrode.**

In a typical method[1,3], 0.24g $Na_2SO_4$ was mixed with 0.4g $FeCl_3{\cdot}H_2O$ and dissolved in

deionised water, and similarly, the as-obtained solution, CN-20 and clean Ni foam were transferred to a 50ml Teflon-lined stainless autoclave for hydrothermal reaction at 120°C for 6 h. After that, the electrodes were taken out and dried in a vacuum oven at 60 °C for overnight. The FeCN electrode was obtained after annealing the as-prepared Ni foams at 450 °C for 3 h with a heating rate of 3 °C/min.

0.84 g $Na_2MoO_4 \cdot 2H_2O$ and 2.1 g sodium salicylate were dissolved in 10 ml deionised water by stirring vigorously. Then, the dispersions were adjusted to pH = 2 by concentrated hydrochloric acid solution. The as-obtained solution, CN-20, and clean Ni foam were transferred to a 50ml Teflon-lined stainless autoclave for hydrothermal reaction at 180°C for 24 h[4]. After that, the electrodes were taken out and dried in a vacuum oven at 60 °C for overnight. The MoCN electrode was obtained by annealing the as-prepared Ni foams at 400 °C for 3 h with a heating rate of 3 °C/min.

**Fabrication of the CoCN//AC ASC**

The CoCN-0.55 electrode and the activated carbon electrode, 3M KOH aqueous electrolyte and a piece of cellulose paper separator were assembled in a CR2016-type coin. The CoCN//AC ASC was obtained after drying at the room temperature.

**Fabrication of the CoCN//CoCN ASSD**

The PVA/KOH gel electrolyte was prepared by dissolving 2.0 g PVA solid and 2.0 g KOH solid in 30 ml 80 °C deionised water. Two pieces of the electrodes were immersed in the electrolyte for 2 min, taken out and assembled with the separator and copper-plated metal fibers.[2] In the end, the symmetrical CoCN//CoCN ASSD was prepared after drying in a vacuum oven at 40°C.

**Electrochemical measurements**

Electrochemical tests including GCD, CV and EIS measurements of the prepared electrodes were conducted on an electrochemical workstation (CH Instruments Inc. 760E) in a typical three-electrode system, with 3 M KOH aqueous electrolyte, Ag/AgCl reference electrode and Pt foil counter electrode. EIS measurements were carried out with an AC amplitude of 5 mV in the 0.1Hz-100 kHz frequency range.

**Electrochemical measurements on PIEC behavior**

CoCN-0.55 electrode was irradiated under the simulated sunlight (300 W Xenon lamp source, PLS-SXE300/300UV, Beijing Perfectlight Technology Co., Ltd.) in a three-electrode system with a quartz electrolytic cell, as shown in **Figure S27**. The two-electrode system

measurements of ASSD were configured, as **Figure S28**.

### Photocatalytic HER tests

The photocatalytic HER tests were performed on an on-line photocatalytic $H_2$ evolution system with a 300 W Xe lamp as the light source at room temperature and vacuum pressure.[5] 50 mg sample was dispersed in 30 ml deionised water. After that, the reactor was irradiated under simulated sunlight for 15 min. In a typical experiment, the mixture and extra 60 ml deionised water were poured into the reactor, then, 10 ml lactic acid was added as the sacrificial agent. The suspension was irradiated by a 300 W Xe lamp as the UV-visible light source. The resulting gases were collected and analyzed by a gas chromatograph equipped with a thermal conductivity detector and a capillary column (GC 7900, Tianmei, Shanghai). The current of the lamp was kept constant in the experiment.

### Photoelectrochemical (PEC) Measurements

PEC measurements including photocurrent density and sensitivity measurements, EIS and Mott-Schottky measurements were performed on the electrochemical working station in a three-electrode system with 0.1M $Na_2SO_4$ aqueous electrolyte, an Ag/AgCl reference electrode and a Pt wire counter electrode. The 300W Xe arc lamp was selected as the light source.

The working electrode was prepared as follows: 10 mg sample was dispersed into 1 ml alcohol and a slurry was obtained. After that, the slurry was dropped on the surface of ITO glass (2.5 cm × 4.5 cm) evenly, the working electrode was prepared after drying for overnight at the room temperature.

### Characterization

XRD was measured on a D8 Advance diffractometer (Bruker, Germany) with Cu Kα radiation ($\lambda$ = 0.15418 nm) at the 2θ range from 10° to 80°. Scanning electron microscopy (SEM) images were collected on a Carl ZEISS-SUPRA55 (Germany) Field emission scanning electron microscope. Transmission electron microscopy (TEM, high-resolution TEM, JEM-2100F) characterization was carried out to confirm the micro-/nano-structure and morphology. Atomic Force Microscope characterization was conducted on a Dimension Icon (Bruker, Germany) scanning with a probe scanning mode. DRS characterization was recorded on a UV-2500 Shimadzu Japan spectrophotometer. X-ray photoelectron spectroscopy (XPS) was conducted to determine the elementary composition and the surface

states on a PerkinElmer PHI 5000C X-ray photoelectron spectroscopy instrument with Al K Alpha as the Source Gun Type. Contact angle measurements were carried out on a JC2000D Contact Angle Analyzer (Shanghai, China). PL emission spectra were obtained on an X-ray fluorescence spectroscopy (Hitachi, F-4600, Japan) with a 150 W Xe lamp as the excitation source, the excited wavelength was chosen as 425 nm. Specific surface area was measured by $N_2$ adsorption/desorption measurements and was analyzed by the BET method on a Micromeritics ASAP 2460 (Autosorb-iQ-2MP) instrument.

**XAFS experiments**

CoCN-0.55 sample was ground for 20 min, 2 mg sample was taken out and mixed with 50 mg Boron Nitride homogeneously, then it was pressed into circular pellets with a diameter of 10 mm and stuck to the tape.

The XAFS measurements were performed at 4B9A experimental station in BSRF. The storage rings of BSRF was operated at 2.5 GeV with a maximum current of 450 mA. Si (111) double-crystal monochromator was used to monochromatize the X-ray beam.[6] XAFS data were collected in transmission at the energy range from ~130 eV to 1,000 eV above the Co K-edge. The measurement error of $\mu(E)$ is less than $10^{-3}$.

Original XAFS data was processed by the Athena software according to the standard procedures. The quantitative curve-fittings in the R-space were performed with Fourier-transformed k-space results by the IFEFFIT module in Artemis software[7]. During the curve-fitting, the overall amplitude reduction factor S02 was a guess value. The fitting range of Co-O and Co-Co shells in the R-space was 1.0-6.0 Å and the k- space was 1.0-15.0 $Å^{-1}$.

# 2. Equations in Text

$$C = \frac{I \times \Delta t}{S \times \Delta V} \quad \textbf{(S1)}$$

where C is the specific capacity, I is the current during testing, (ΔV/Δt) is the voltage scan rate, S is the total area of the electrodes.[8,9] (S = 1 $cm^2$ in this work)

$$C = \frac{\int I du}{2v\ (2m)\ \Delta V} \times 4 \quad \textbf{(S2)}$$

where the denominator is the curve area in CV curve, $v$, $m$, and $\Delta V$ is scan rate (mV $s^{-1}$), the mass of active material (g), and potential range (V), respectively.[10]

$$Z_{Re} = R_s + R_{ct} + \sigma_W \omega^{-1/2} \quad \textbf{(S3)}$$

$$D = \frac{R^2T^2}{2A^2N^4F^4C^2\sigma_W^2} \quad \textbf{(S4)}$$

where $Z_{Re}$, $R_S$, $R_{ct}$, $R$, $T$, $A$, $N$, $F$, $C$, $\sigma_W$ are the real resistance, electrolyte resistance, charge transfer resistance, ideal gas constant, temperature, electrode area, reacting electron numbers, Faraday constant, electrolyte concentration and Warburg coefficient, respectively.[11,12]

$$E = \frac{C\Delta V^2}{2 \times 3.6} \quad \textbf{(S5)}$$

$$P = 3600\frac{E}{\Delta t} \quad \textbf{(S6)}$$

where $C$ (F $cm^{-2}$) is area capacity, $\Delta V$ (V) is the working potential of the device, $\Delta t$ (s) is the discharging time.[13]

$$C = \frac{Q}{V} \quad \textbf{(S7)}$$

where $Q$ is the quantity of electric charge, $V$ is the potential.[8]

$$E = \frac{1}{2}CV^2 \quad \textbf{(S8)}$$

where $V$ is the working voltage.[14]

# 3. Supplemental Figures and Tables

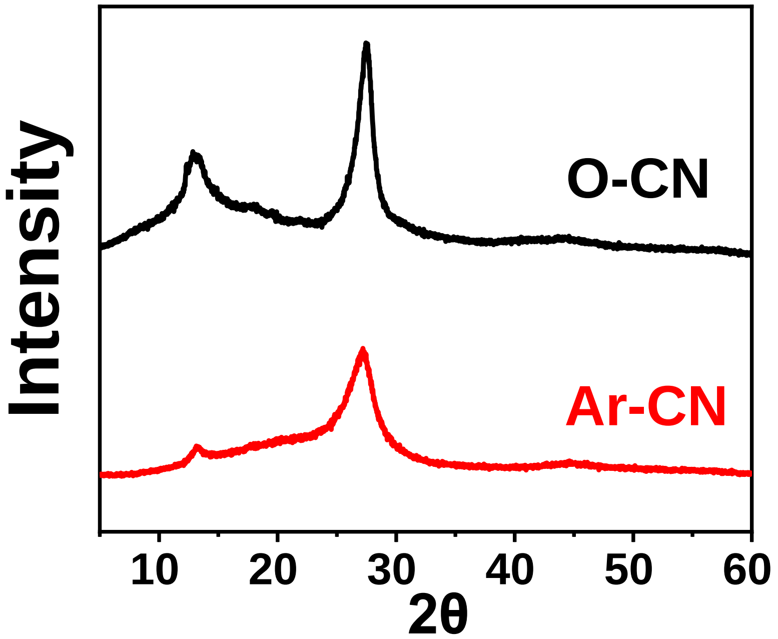


**Figure S1** | XRD patterns of g-$C_3N_4$ calcined in air (O-CN) and argon (Ar-CN).

**Notes:**

The 13.1° peak of Ar-g-$C_3N_4$ almost disappears, which is related to the hydrogen bond that maintains the long-range atomic order in the interlayer, indicating that the hydrogen bond is broken after heating[15]. Also, the intensity of the 27.2° peak of Ar-CN decreased obviously, indicating that the crystallinity of g-$C_3N_4$ was decreased[16].

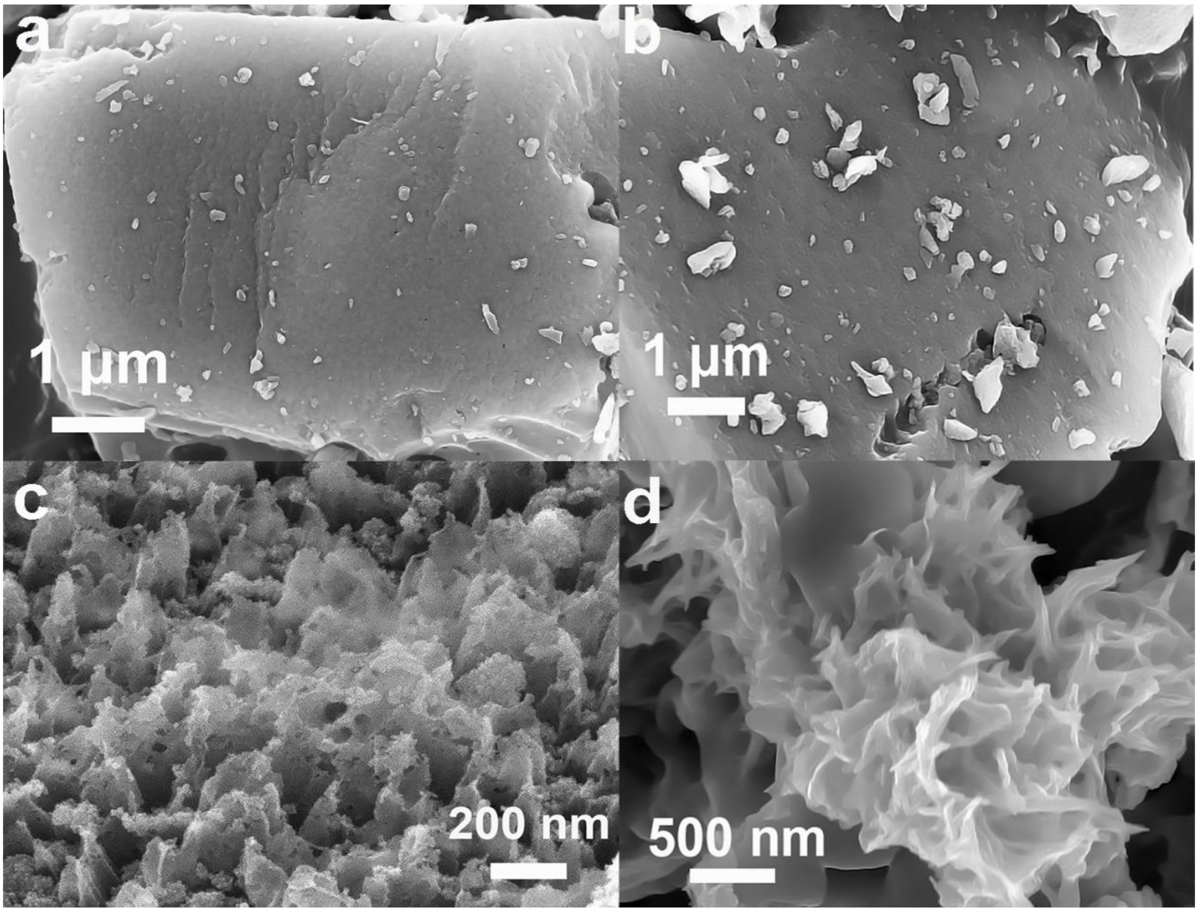


**Figure S2 |** SEM images of **a** CoCN-0.14, **b** CoCN-0.28, **c** CoCN-0.55 and **d** CoCN-1.1 samples.

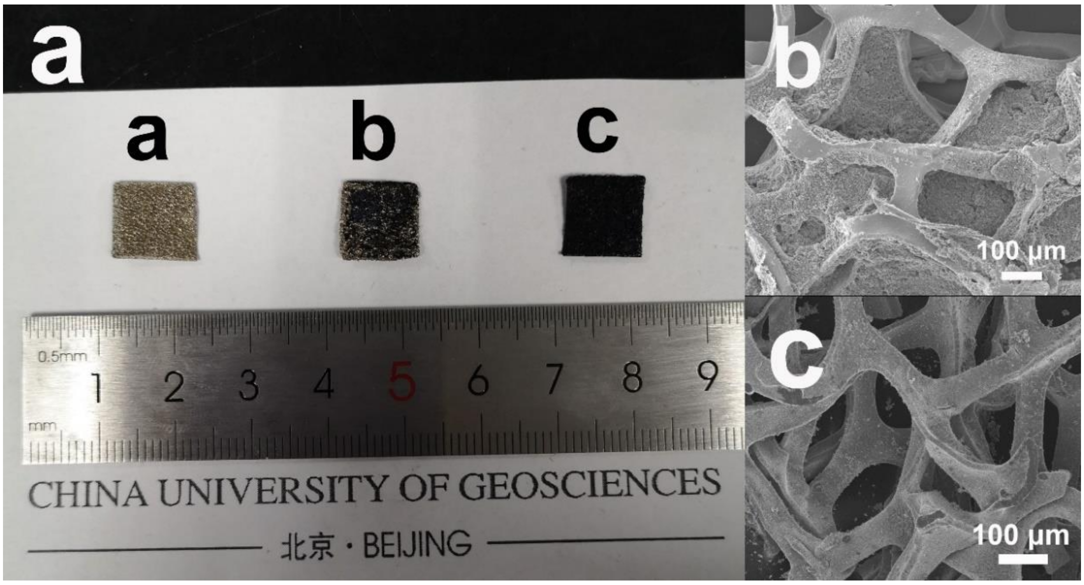


**Figure S3 |** a) Image of **a** nickel foam, **b** CoCN-0.55 electrode prepared by traditional process and **c** CoCN-0.55 electrode in this work, b) SEM image of **b**, c) SEM image of **c**.

**Note:** Tranditional process was: The CoCN-0.55 samples, acetylene black and polyvinylidene fluoride (PVDF) were mixed in an N-methyl-2-pyrrolidone (NMP) solvent with a weight ratio of 8: 1: 1 and formed as a slurry, and then the slurry was stuck on clean Ni foam and dried in a vacuum oven at 80°C for overnight.

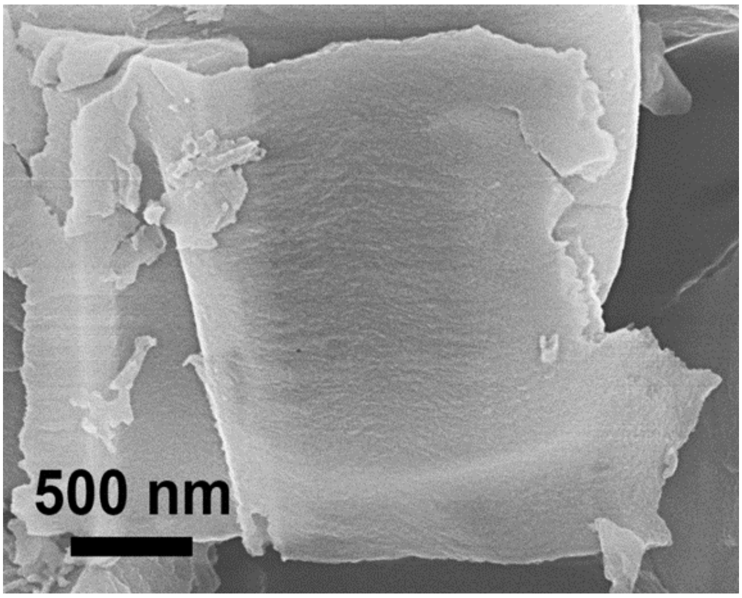


**Figure S4 |** SEM image of g-$C_3N_4$ calcined in Ar atmosphere.

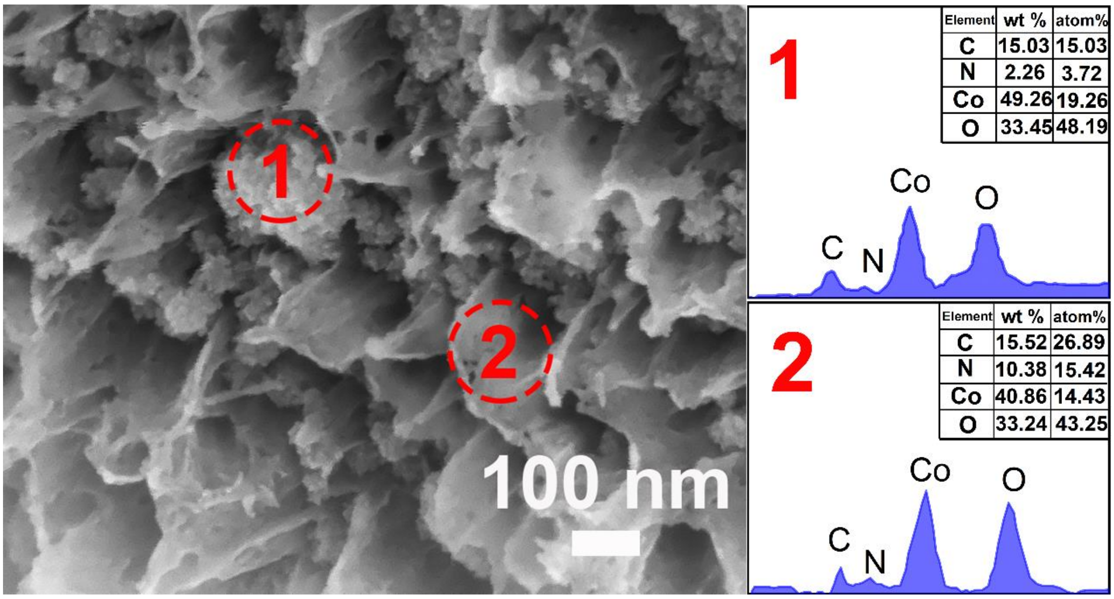


**Figure S5 |** SEM image of the CoCN-0.55 sample. Insets 1 and 2 are the proportions of the relevant elements.

Co distribution at region 1 is higher than that at region 2, N distribution at region 1 is lower than that at region 2, revealing the nanoparticles at region 1 may be $Co_3O_4$.

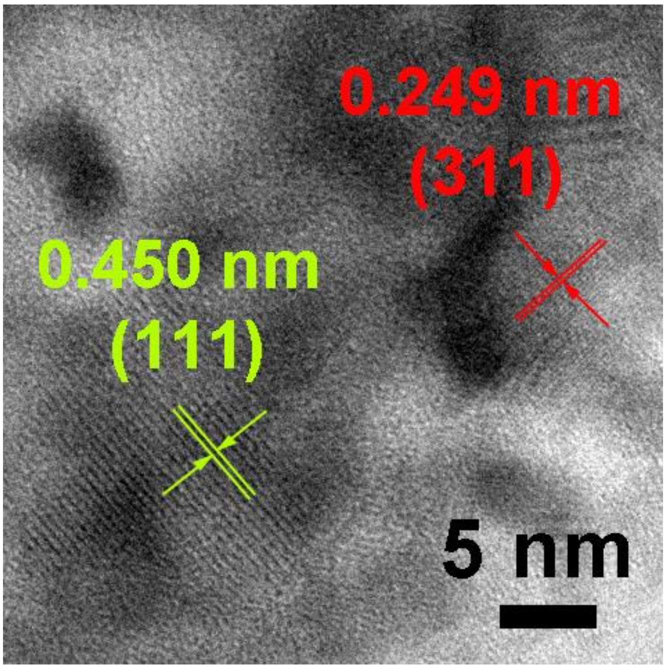


**Figure S6 |** HRTEM image of CoCN-0.55 sample.

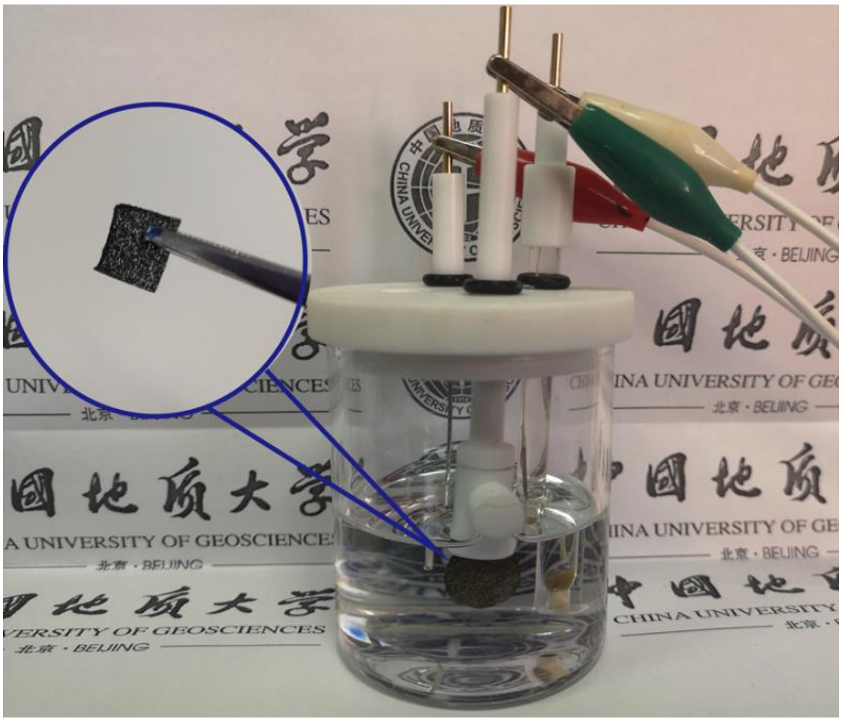


**Figure S7 |** The three-electrode system for related electrochemical measurement.

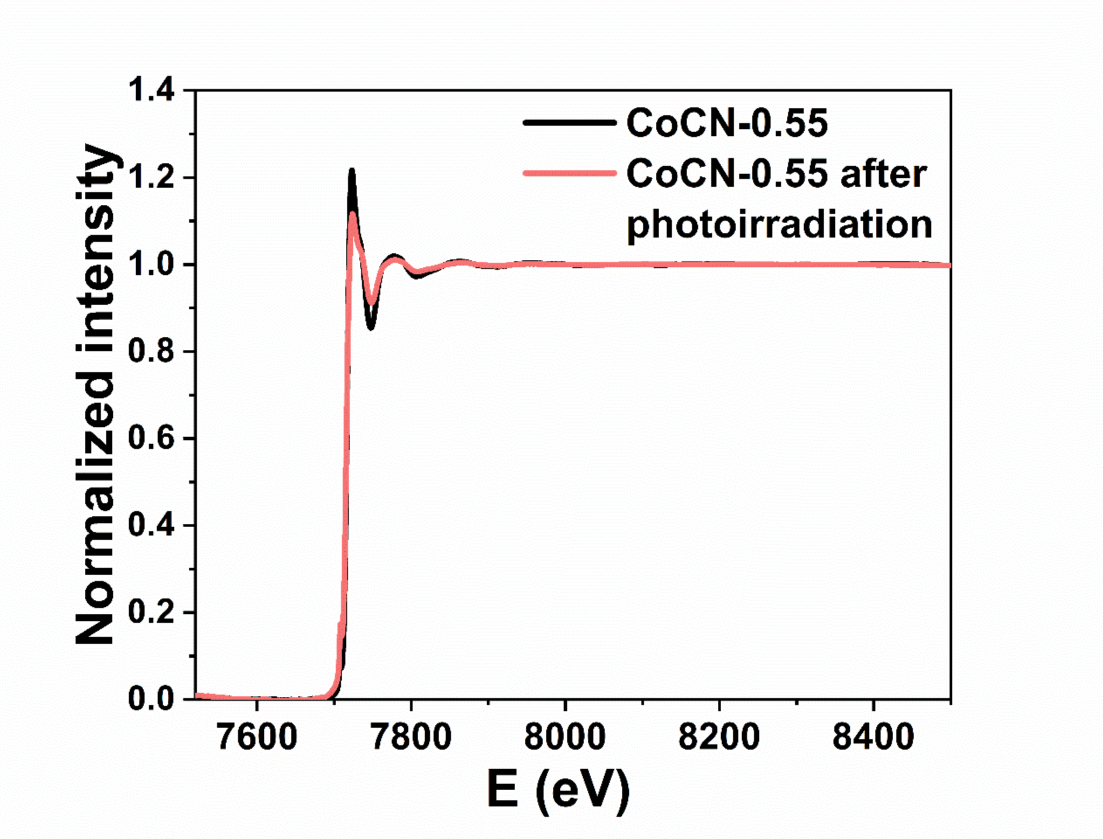


**Figure S8** | Co K-edge XAFS spectra of CoCN-0.55 sample before and after photoirradiation.

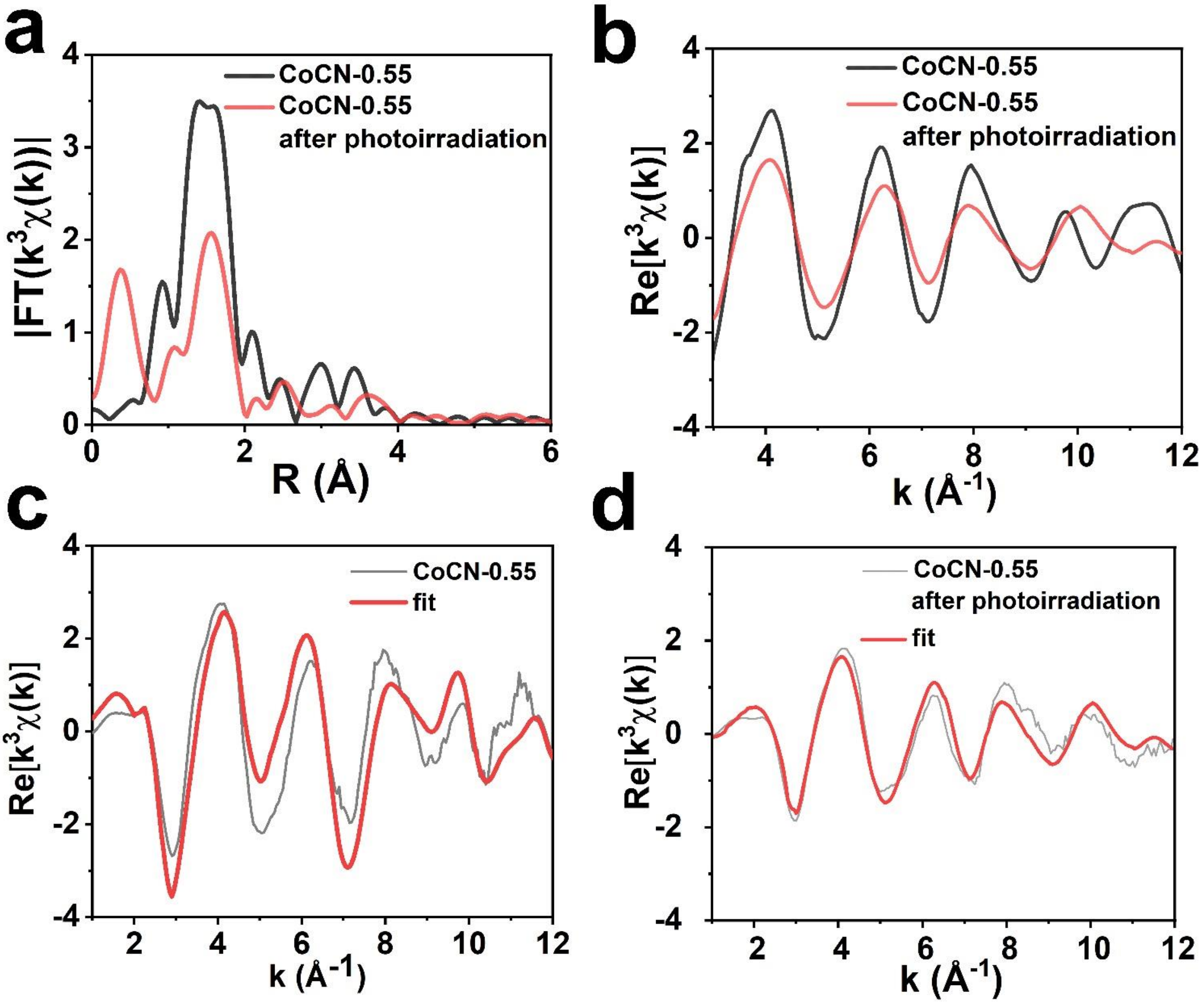


**Figure S9 |** Curve fitting results of the EXAFS: $k^3\chi(k)$ Fourier transform spectra.

**Table S1** | EXAFS curve-fitting results.

| Sample | Path | N | R (Å) | $\sigma^2$ ($10^{-3}$Å$^2$) |
|---|---|---|---|---|
| $Co_3O_4$ [6,17] | Co-O<br>Co-$O_1$<br>Co-$O_2$<br>Co-$Co_1$<br>Co-$Co_2$ | 5.3<br>5.3<br>4.0<br>4.0<br>9.3 | 1.91<br>3.26<br>3.56<br>2.85<br>3.37 | – |
| **CoCN-0.55** | Co-O<br>Co-$O_1$<br>Co-$O_2$<br>Co-$Co_1$<br>Co-$Co_2$ | 4.0±0.2<br>4.0±0.2<br>6.0±0.3<br>4.0±0.2<br>12.0±0.2 | 2.28±0.01<br>1.86±0.01<br>1.86±0.01<br>3.64±0.01<br>3.27±0.01 | 4.2±0.1<br>3.1±0.1<br>3.1±0.1<br>8.0±0.1<br>2.8±0.1 |
| **CoCN-0.55 after photoirradiation** | Co-O<br>Co-$O_1$<br>Co-$O_2$<br>Co-$Co_1$<br>Co-$Co_2$ | 4.0±0.2<br>4.0±0.2<br>6.0±0.2<br>4.0±0.2<br>12.0±0.2 | 2.06±0.01<br>1.17±0.02<br>0.90±0.02<br>3.49±0.01<br>3.36±0.01 | 1.6±0.1<br>2.0±0.2<br>0.8±0.1<br>3.1±0.2<br>3.5±0.2 |

**Note:** The internal atomic distance is R (Å); Debye-Waller factor is $\sigma^2$ ($10^{-3}$Å$^2$).

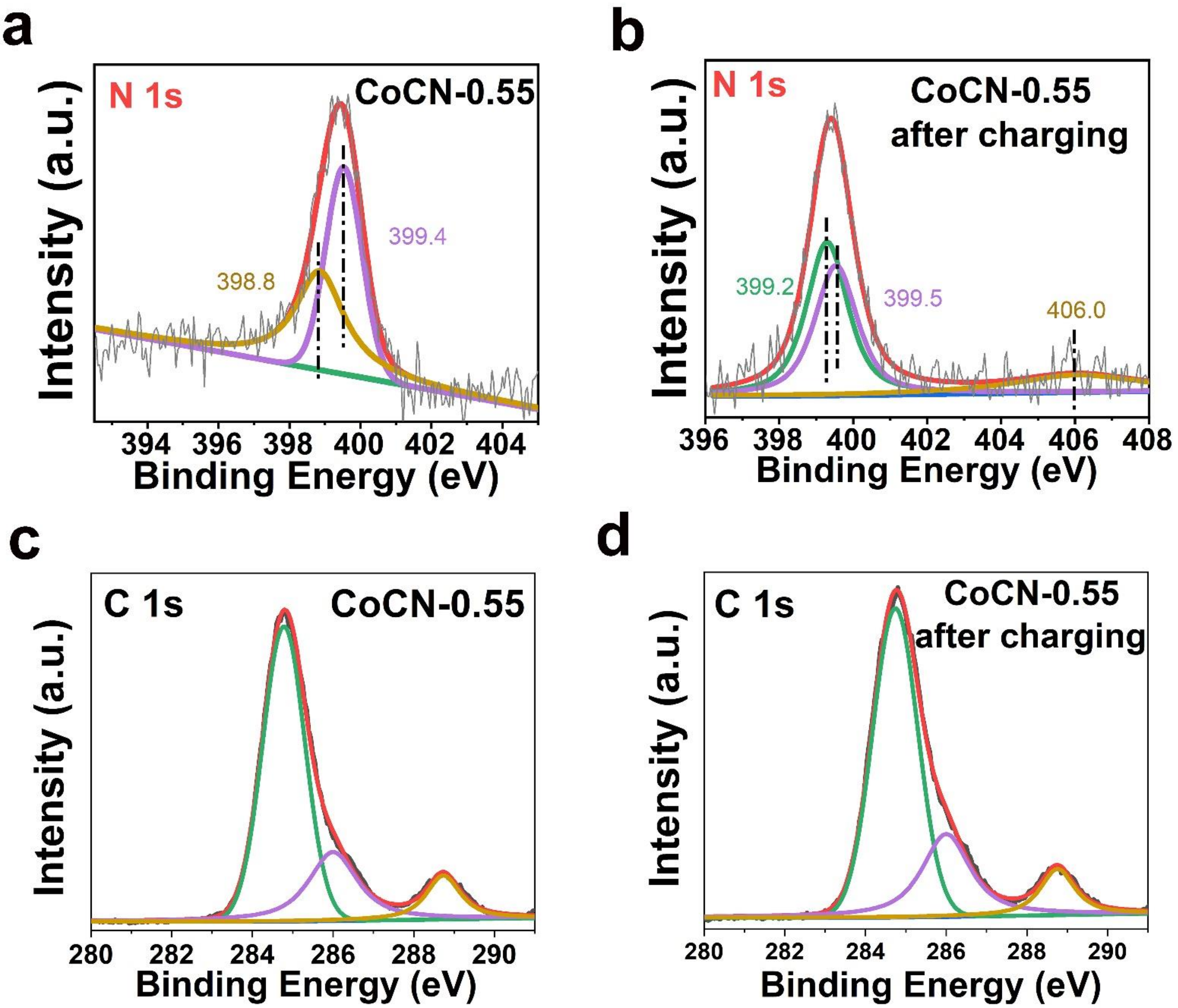


**Figure S10** N 1s peaks of (a) CoCN-0.55 and (b) CoCN-0.55 after charging, and C 1s peaks of (c) CoCN-0.55 and (d) CoCN-0.55 after charging.

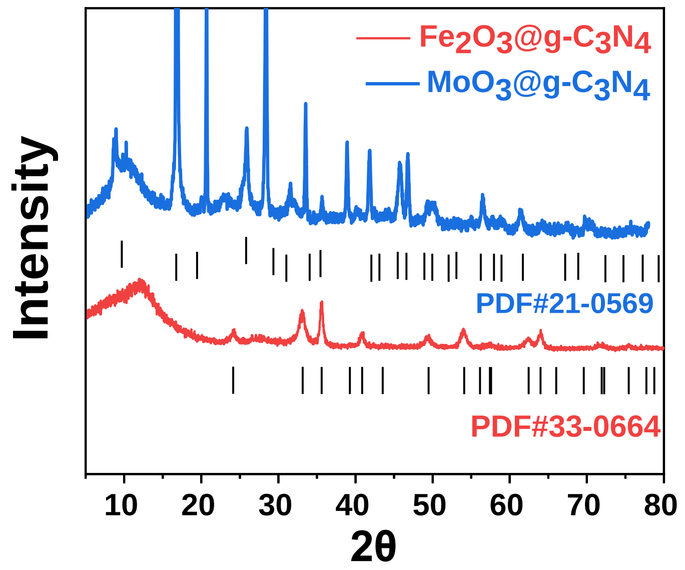


**Figure S11** XRD patterns of FeCN and MoCN samples, and the corresponding PDF cards: JCPDS #21-0569 ($Fe_2O_3$ phase) and #33-0664 ($MoO_3$ phase).

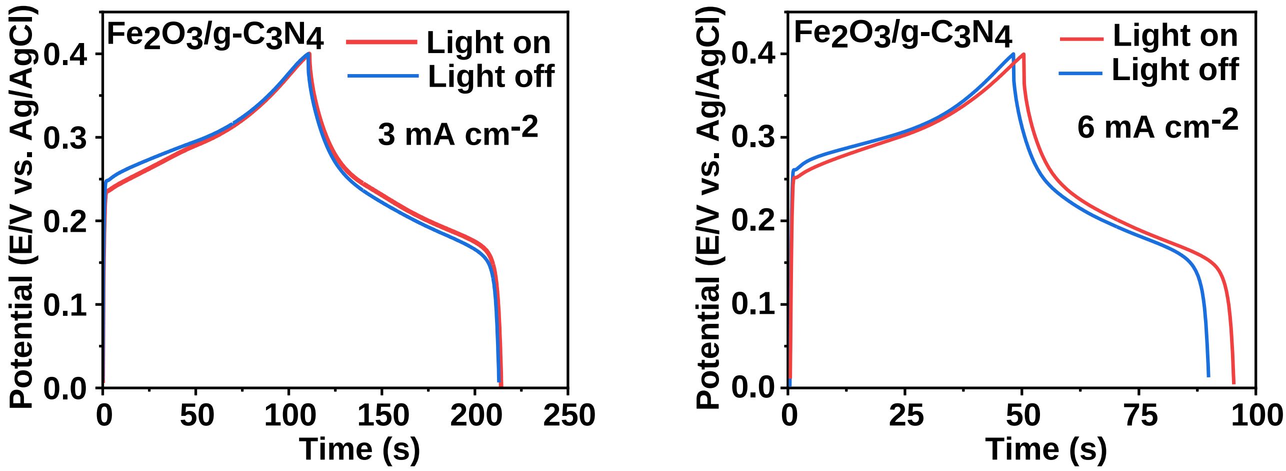


**Figure S12** GCD curves of FeCN electrode at the current density of 3 mA $cm^{-2}$ (Left) and 6 mA $cm^{-2}$ (Right) without (blue curve) and with photoirradiation (red curve).

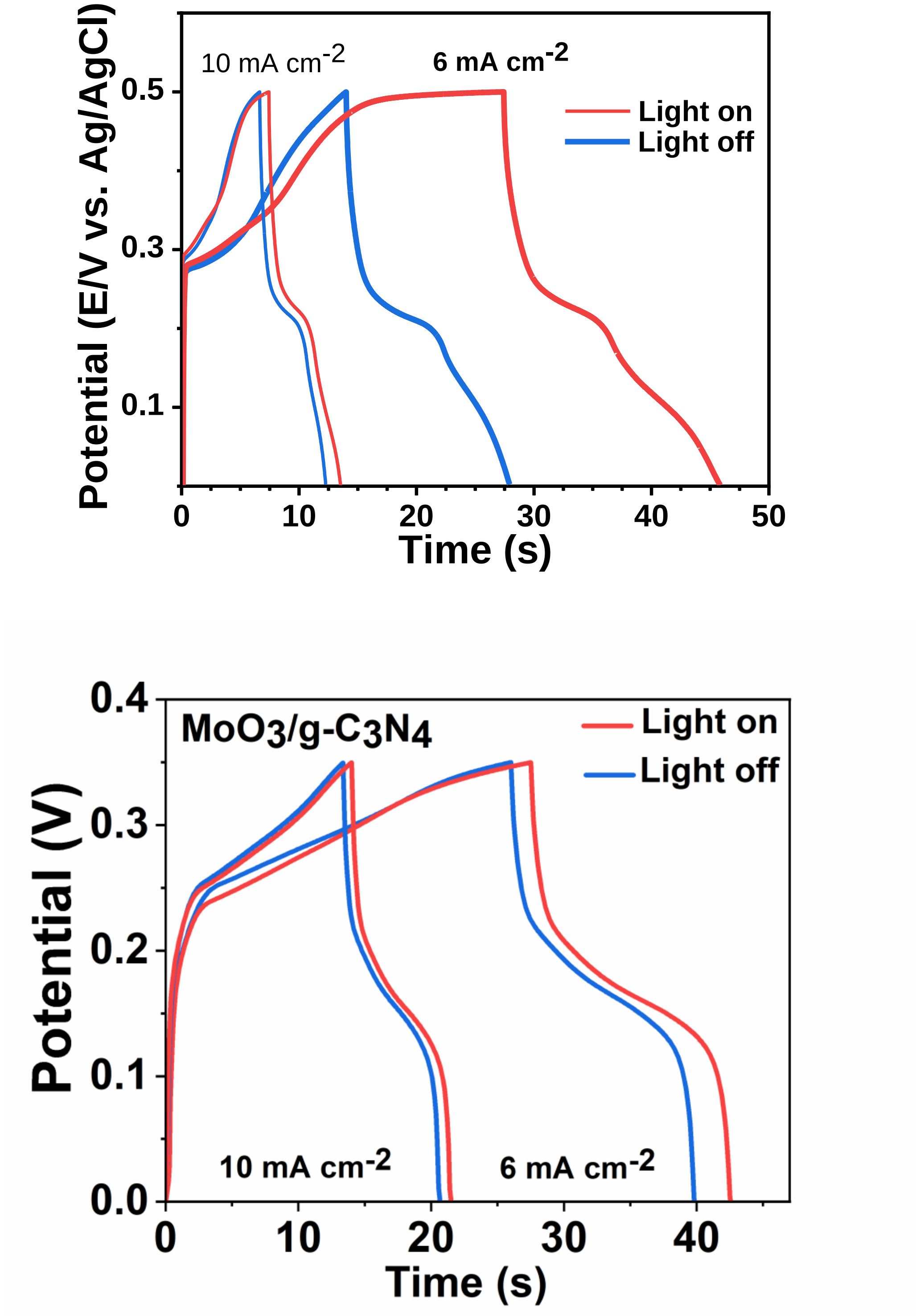


**Figure S13** GCD curves of g-$C_3N_4$ (Up), MoCN (Down) electrode at the current density of 6 mA cm$^{-2}$ and 10 mA cm$^{-2}$ without (blue curve) and without photoirradiation (red curve).

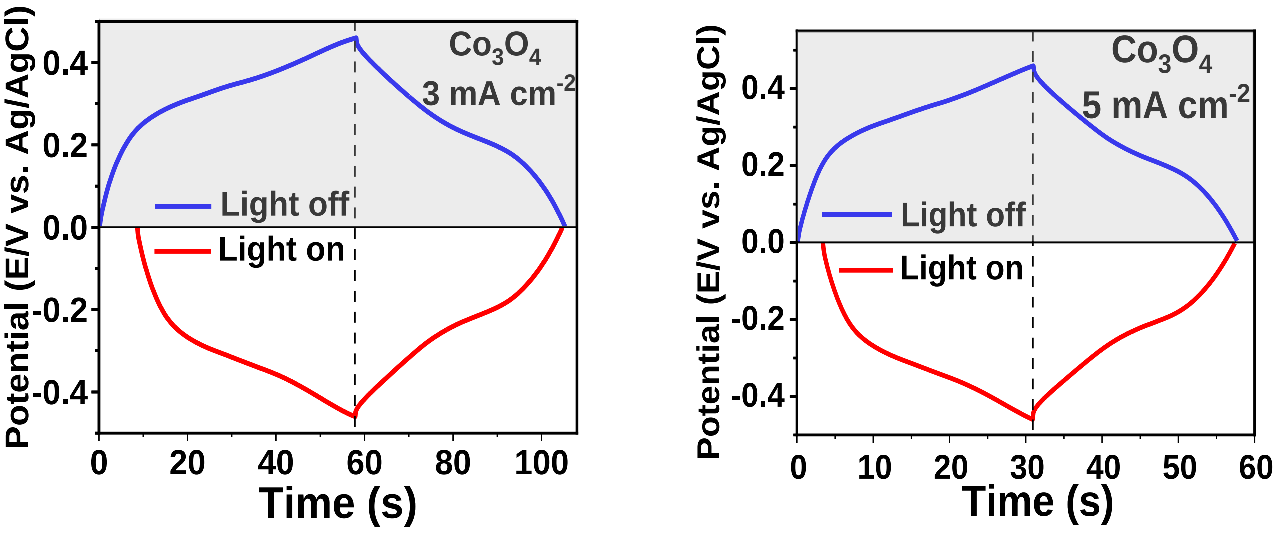


**Figure S14** GCD curves of $Co_3O_4$ electrode at the current density of 3 mA $cm^{-2}$ (Left) and 5 mA $cm^{-2}$ (Right) without (blue curve) and with photoirradiation (red curve).

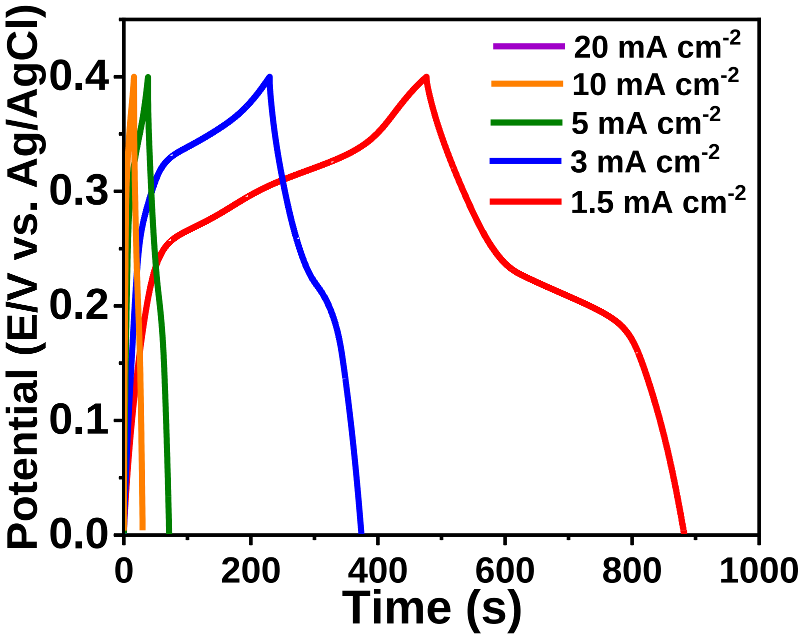


**Figure S15** GCD curves of CoCN-0.55 electrode measured in three-electrode system.

**Table S2** GCD capacity of related electrodes with and without photoirradiation at the current density of 6 mA $cm^{-2}$.

| Treatment / Samples | Without photoirradiation (mF $cm^{-2}$) | With photoirradiation (mF $cm^{-2}$) |
|---|---|---|
| **$Co_3O_4$** | 288.0 | 290.2 |
| **g-$C_3N_4$** | 167.3 | 220.4 |
| **MoCN** | 234.9 | 257.1 |
| **FeCN** | 625.5 | 673.5 |
| **CoCN-0.55** | 943.8 | 1088.0 |

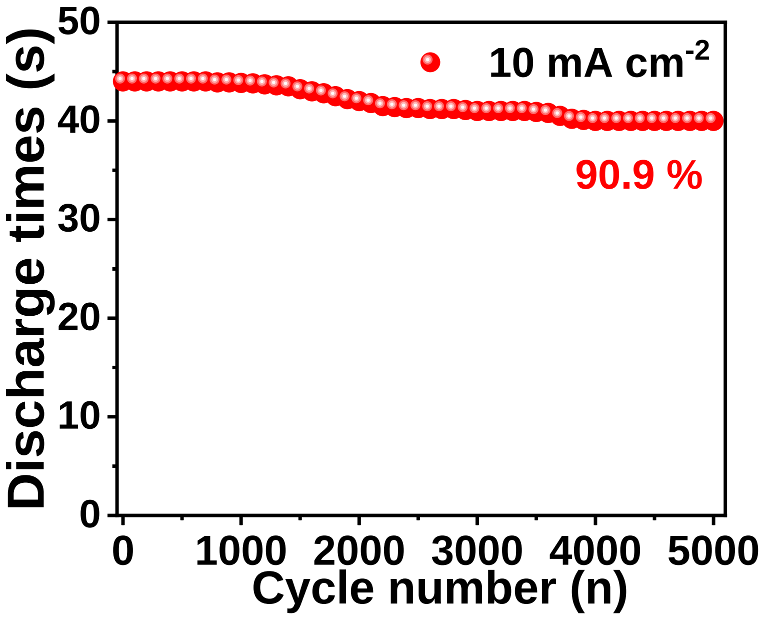


**Figure S16** Cycling performance of the CoCN-0.55 electrode at a current density of 10 mA $cm^{-2}$ .

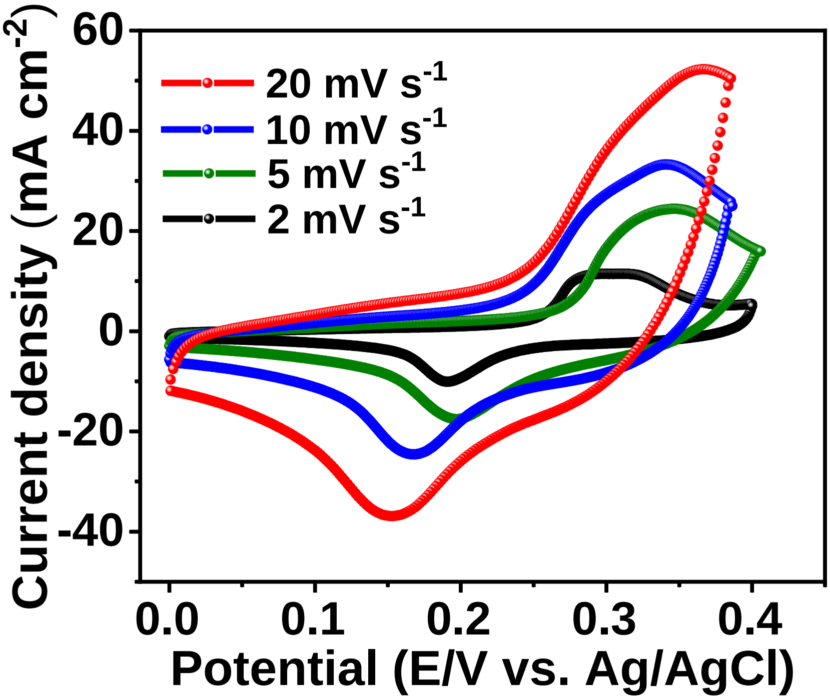


**Figure S17** CV curve of CoCN-0.55 electrode with scanning rate of 2-20 mV $s^{-1}$.

**Table S3** GCD capacity of the CoCN-0.55 electrode.

| Current density (mA $cm^{-2}$) | Discharge times (s) | Capacity (mF $cm^{-2}$) | Current density (A $g^{-1}$) | Capacity (F $g^{-1}$) |
|---|---|---|---|---|
| 1.5 | 405.2 | 1519.5 | 1.5 | 1520 |
| 3.0 | 144.4 | 1083.0 | 3 | 1083 |
| 5.0 | 75.5 | 943.8 | 5 | 944 |
| 10.0 | 33.3 | 832.5 | 10 | 833 |
| 20.0 | 13.5 | 675.0 | 20 | 675 |

**Table S4** The comparison of the GCD capacity of some $Co_3O_4$-based composites, carbon-based composites and g-$C_3N_4$-based composites reported in literatures.

| Electrode material | Electrolyte | Discharge current density | Specific capacity | Rate capability (current density) | Ref. |
|---|---|---|---|---|---|
| $Co_3O_4$/g-$C_3N_4$ | KOH | 1.5 mA $cm^{-2}$<br>1.5 A $g^{-1}$<br>20 mA $cm^{-2}$<br>20 A $g^{-1}$ | 1.52 F $cm^{-2}$<br>1519.5 F $g^{-1}$<br>0.68 F $cm^{-2}$<br>675 F $g^{-1}$ | 5000 cycles<br>90.9%<br>(10 mA $cm^{-2}$) | **This work** |
| NiO/$Co_3O_4$/GQDs | KOH | 1 A $g^{-1}$ | 1361 F $g^{-1}$ | 3000 cycles,<br>76.4%<br>(10 A $g^{-1}$) | [18] |
| Pd-$Co_3O_4$<br>(oxygen-vacancy-rich $Co_3O_4$) | KOH | 7 mA $cm^{-2}$ | 4.7 F $cm^{-2}$<br>(1353 F $g^{-1}$)<br>Mass loading： | 5000 cycles,<br>95%<br>(7 mA $cm^{-2}$) | [19] |
| Oxygen-vacancy<br>$Co_3O_4$/graphene | KOH | 1 A $g^{-1}$ | 978.1 F $g^{-1}$ | 93.7%<br>( 10 A $g^{-1}$ ) | [20] |
| sandwich-structured<br>$Co_3O_4$/Carbon Fiber/$Co_3O_4$ | KOH | 0.5 A $g^{-1}$ | 892 F $g^{-1}$ | 6000 cycles,<br>88% | [21] |
| $Co_3O_4$/nitrogen-doped carbon hollow spheres | KOH | 1 A $g^{-1}$ | 581 F $g^{-1}$ | 91.6%<br>20 A $g^{-1}$ | [22] |

**Note:**

The specific capacity of the CoCN-0.55 *p-n* junction electrode can reach 1519.5 F $g^{-1}$ at the current density of 1.5A $g^{-1}$, which is higher than that of the reported $Co_3O_4$ electrodes and $Co_3O_4$/C composites electrodes (such as N-doped carbon hollow spheres[22], graphene[20], etc.) at the current density of 1 A $g^{-1}$.

In addition, the CoCN-0.55 electrode also has considerable cyclic stability due to the formed *p-n* junction interface and the *in-situ* growth of the active material on the Ni foam, which is more robust than that of the $Co_3O_4$ electrodes but is not as stable as the above-mentioned $Co_3O_4$/C composites, attributing to the good mechanical properties and electrochemical stability of the carbonaceous materials.

**Table S5** GCD capacity of CoCN-0.55 electrode with and without photoirradiation.

| Current density (mA cm$^{-2}$) | Discharge time without photoirradiation (s) | Discharge time without photoirradiation (s) | With photoirradiation (mF cm$^{-2}$) | With photoirradiation (mF cm$^{-2}$) | Current density (A g$^{-1}$) | With photoirradiation (F g$^{-1}$) | With photoirradiation (F g$^{-1}$) |
|---|---|---|---|---|---|---|---|
| 3.0 | 147.3 | 150.3 | 1104.8 | 1127.3 | 3 | 1105 | 1127 |
| 5.0 | 75.5 | 87.1 | 943.8 | 1088.8 | 5 | 944 | 1089 |
| 8.0 | 43.5 | 52.4 | 870.0 | 1048.0 | 8 | 870 | 1048 |
| 20.0 | 12.4 | 16.1 | 620.0 | 805.0 | 20 | 620 | 805 |

**Table S6** Original GCD test data under continuous photoirradiation at the current density of 20 mA $cm^{-2}$.

| Time (s) at 0 V | Time (s) at 0.40 V | Charge time (s) | Discharge time (s) |
|---|---|---|---|
| — | 16.0 | — | 11.4 |
| 27.4 | 39.1 | 11.7 | 11.4 |
| 50.5 | 62.2 | 11.7 | 11.5 |
| 73.7 | 85.4 | 11.7 | 11.5 |
| 96.9 | 109.1 | 12.2 | 11.6 |
| 120.7 | 132.4 | 11.7 | 11.5 |
| 143.9 | 155.6 | 11.7 | 11.6 |
| 167.2 | 178.9 | 11.7 | 11.6 |
| 190.5 | 202.2 | 11.7 | 11.6 |
| 213.8 | 225.5 | 11.7 | 11.6 |
| 237.1 | 248.9 | 11.8 | 11.6 |
| 260.5 | 272.4 | 11.9 | 11.6 |
| 284 | 295.9 | 11.9 | 11.6 |
| 307.5 | 319.5 | 12.0 | 11.5 |
| 331 | 342.9 | 11.9 | 11.7 |
| 354.6 | 366.5 | 11.9 | 11.6 |
| 378.1 | 389.9 | 11.8 | 11.6 |
| 401.5 | 413.4 | 11.9 | 11.6 |
| 425 | 436.9 | 11.9 | 11.6 |
| 448.5 | 460.4 | 11.9 | 11.6 |
| 472 | 483.9 | 11.9 | 11.8 |
| 495.7 | 507.6 | 11.9 | 11.8 |
| 519.4 | 531.3 | 11.9 | 11.8 |
| … | … | … | … |
| 1193.6 | 1205.7 | 12.1 | 12.1 |
| 1217.8 | 1230 | 12.2 | 12.1 |
| 1242.1 | 1254.3 | 12.2 | 12.1 |
| … | … | … | … |
| 1461.7 | 1474 | 12.3 | 12.2 |
| 1486.2 | 1498.5 | 12.3 | 12.2 |
| 1510.7 | 1523 | 12.3 | 12.2 |
| … | … | … | … |
| 2102 | 2114.4 | 12.4 | 12.4 |
| 2126.8 | 2139.3 | 12.5 | 12.4 |
| … | … | … | … |
| 2151.7 | 2164.2 | 12.5 | 12.4 |

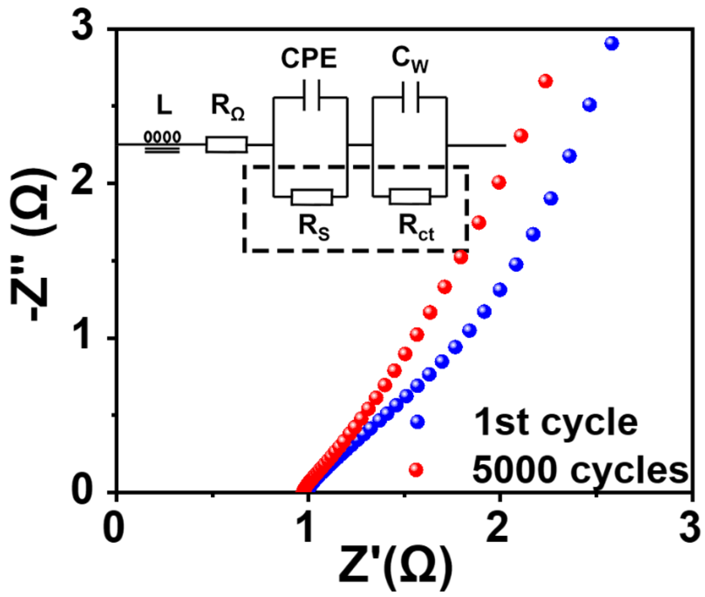


**Figure S18** EIS curves after the first GCD and 5000 GCD cycles at the high-frequency region.

**Note:**

The existence of ***L*** at low frequency may be due to the coating adsorption and desorption or the electrode partial corrosion[23]. The impedance diagram at the low and high-frequency region is similar to the flattened semicircle, which is the result of the charge transfer process. Thus, the contribution of $\boldsymbol{R_S}$ and $\boldsymbol{R_{ct}}$ should be taken into consideration. ($\boldsymbol{R_S}$ is the interface contact resistance between the material and the collector, and $\boldsymbol{R_\Omega}$ is the combined internal resistance of the electrolyte resistance and the intrinsic resistance of the collector, which can be obtained from the intersection of the high-frequency region of the Nyquist curve on the real axis. The semicircular diameter $\boldsymbol{R_{ct}}$ is the interface charge transfer resistance as well as the resistance of electrochemical reaction on the surface of the electrode. Besides, due to the double-layer structure, the contribution of semicircle may including phase element (**CPE**). [24]

The low-frequency region is an 45° straight line approximately. It cannot be equal to 45° ultimately due to the state variable ***L***. That is to say, it is a diffusion process. In the medium-frequency region, the inclined line describes the transport and diffusion of electrons and ions in the pores of electrode materials. [11] Therefore, it is necessary to calculate the contribution of the Warburg impedance caused by concentration polarization, because Warburg impedance could give the diffusion coefficient for the analysis of the diffusion process.

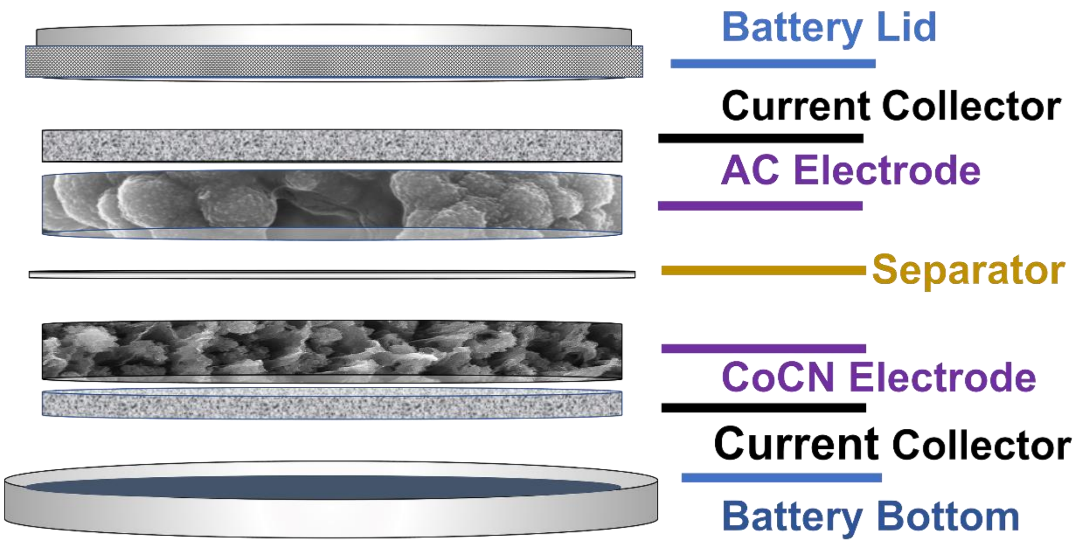


**Figure S19** Schematic diagram of the CoCN//AC supercapacitor device.

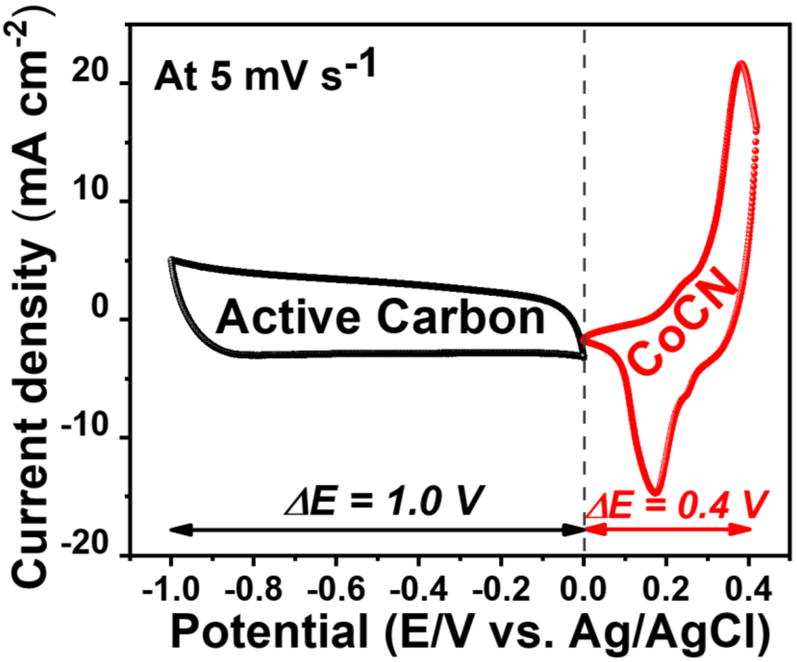


**Figure S20** Voltage contribution of CoCN//AC supercapacitor device. (CV curves were scanned at 5 mV $s^{-1}$)

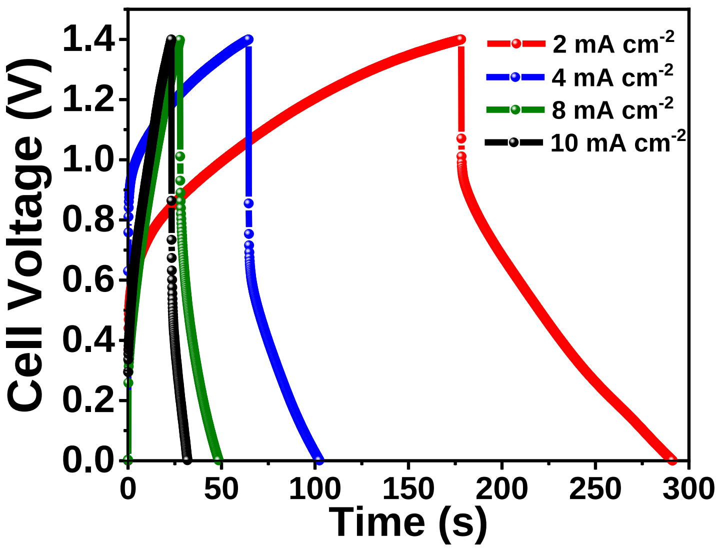


**Figure S21** GCD curves of the CoCN//AC supercapacitor device at the current densities from 2 mA $cm^{-2}$ to 10 mA $cm^{-2}$.

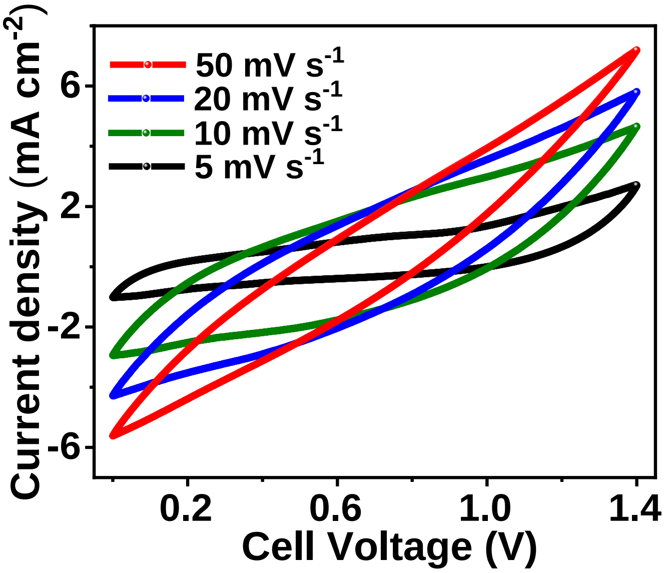


**Figure S22** CV curves of the AC//CoCN supercapacitor device.

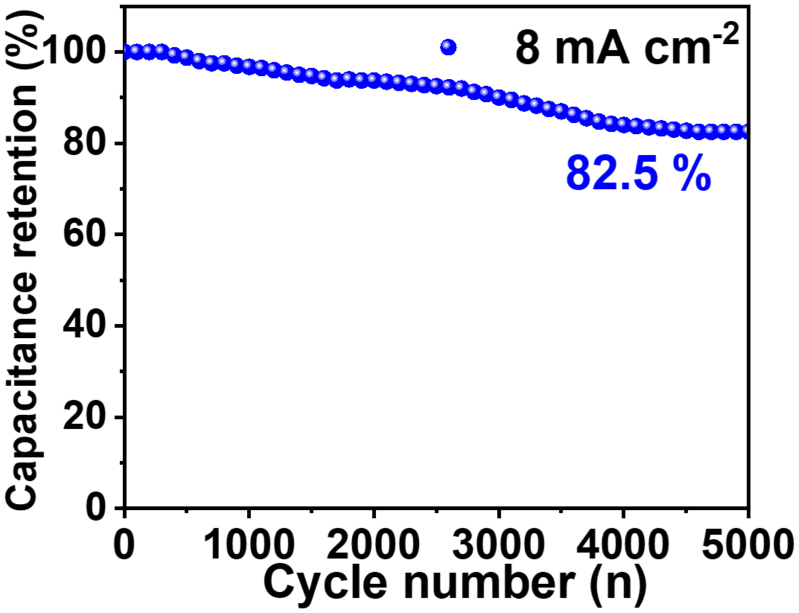


**Figure S23** Cycling performance of the CoCN//AC supercapacitor device.

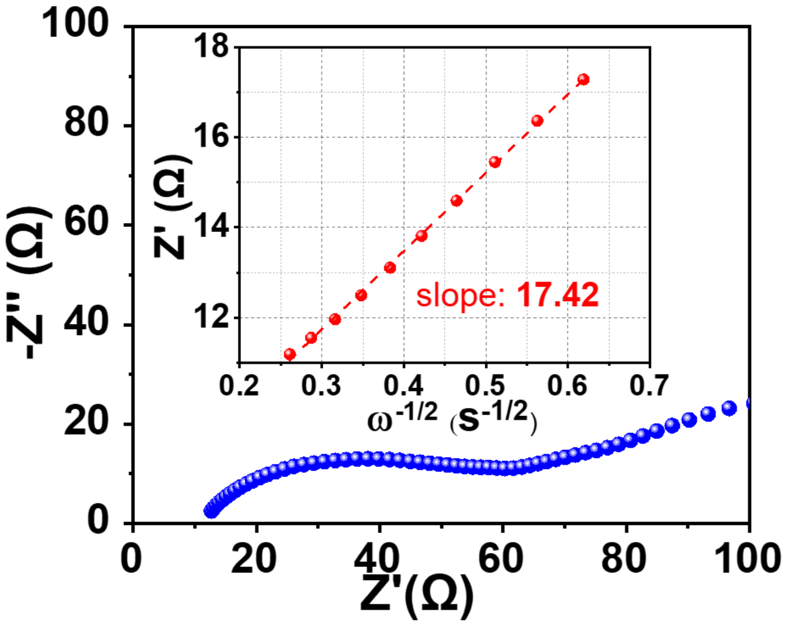


**Figure S24** Nyquist plots of the CoCN//AC supercapacitor device, the inset diagram indicates the fitted $Z_{Re}$-$\omega^{-1/2}$ curve at the low-frequency region.

**Note:**

The corresponding ion diffusion coefficient ***D*** of the device was calculated to be $2.54\times10^{-12}$ $cm^2$ $s^{-1}$. (The diffusion coefficient of commercial lithium-ion batteries is $10^{-12}$~$10^{-16}$ $cm^2$ $s^{-1}$.)

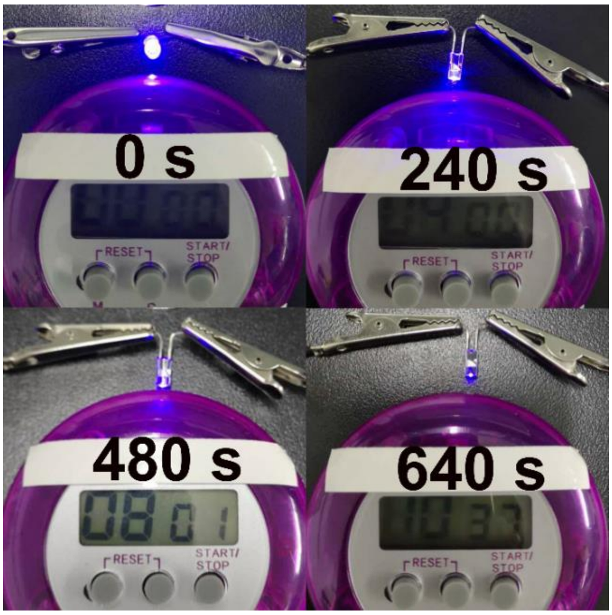


**Figure S25** Demonstration of two CoCN//AC supercapacitor devices in-series, which lighted a LED for more than 10 minutes.

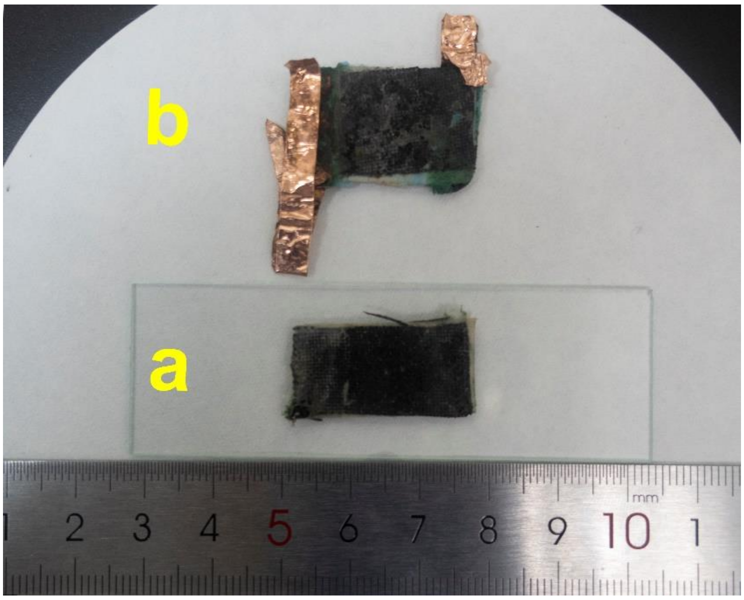


**Figure S26** The image of the CoCN//CoCN ASSD a) before and b) after 5000 GCD cycles.

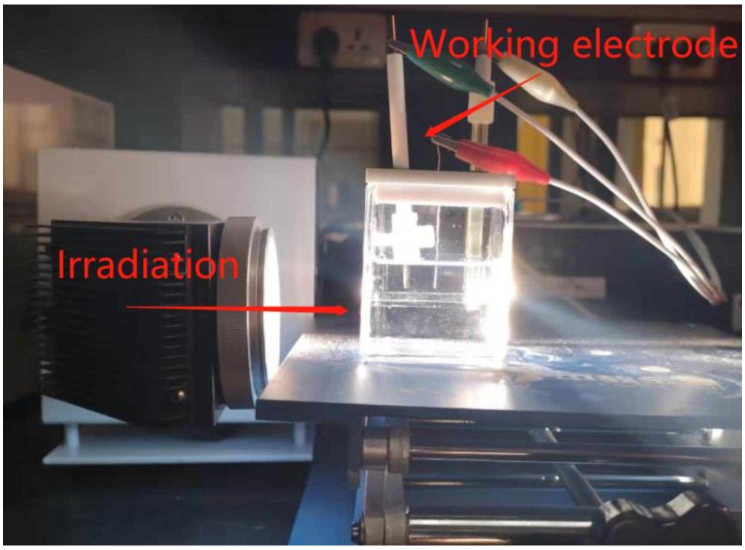


**Figure S27** The measurement of the PIEC behavior of the CoCN-0.55 electrode in three-electrode system.

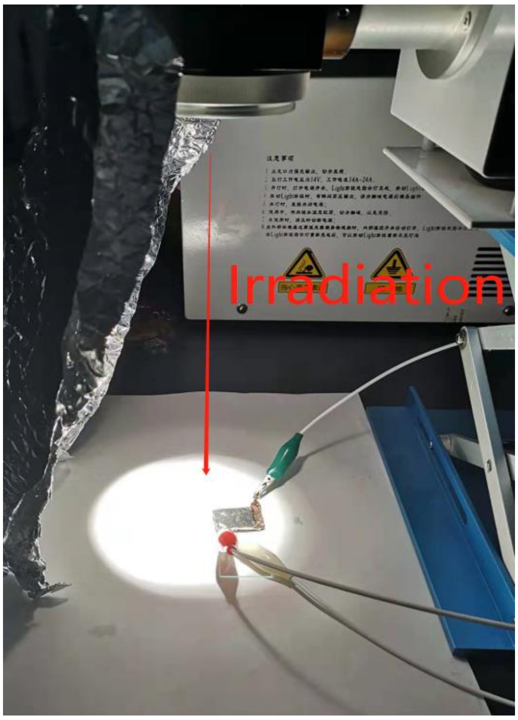


**Figure S28** The measurement of the PIEC behavior of the ASSD in two-electrode system.

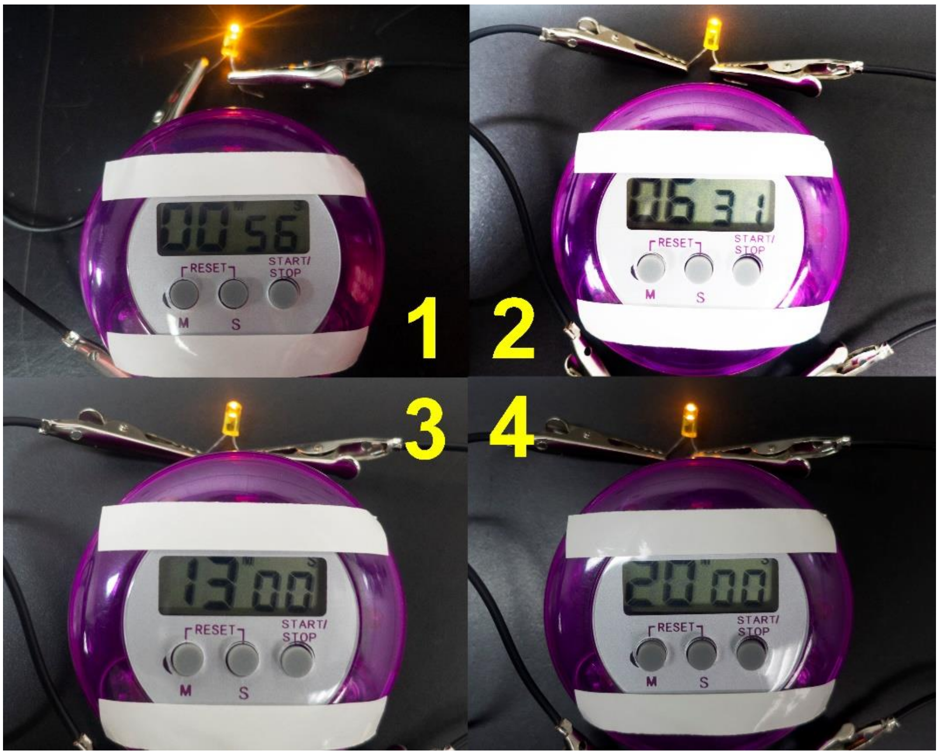


**Figure S29** Demonstration of two CoCN//CoCN ASSDs in-series, which lighted a LED for more than 20 minutes.

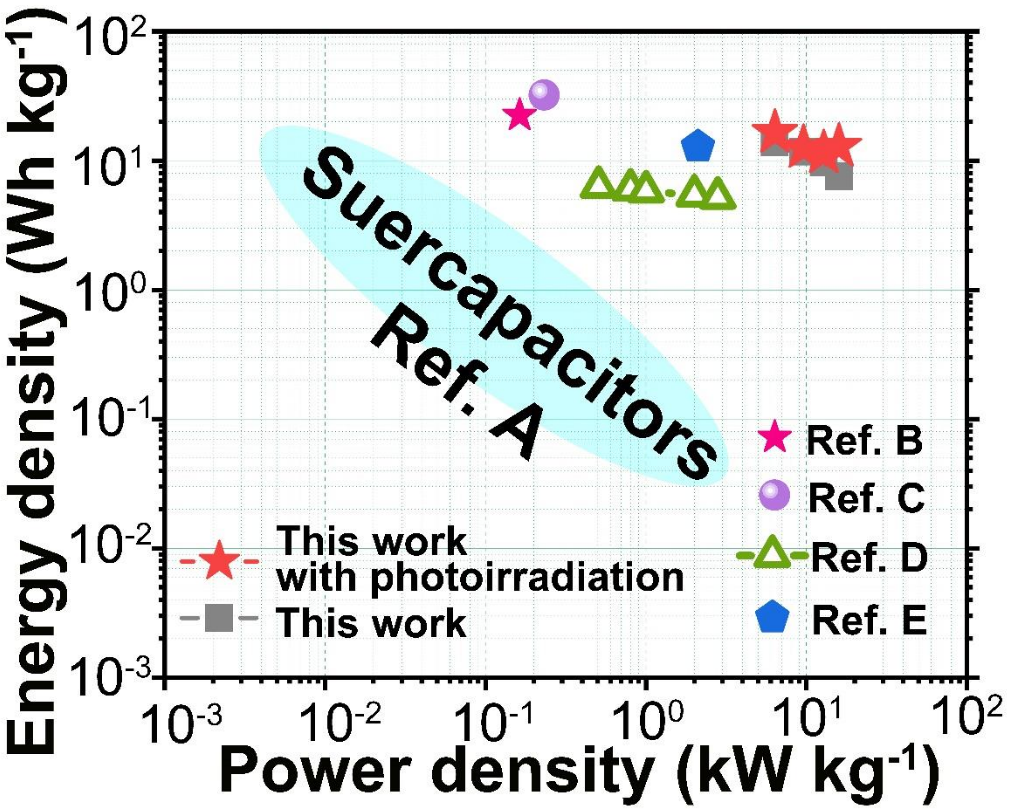


**Figure S30** Ragone plot of the PIEC CoCN//CoCN flexible devices.

**Ref A:** Commercial supercapacitors[8],

**Ref B:** $NiCo_2O_4$//$FeSe_2$ ASC[25],

**Ref C:** $Ni_2CoS_4$@$NiCo_2O_4$//nitrogen-doped carbon xerogels ASC[9],

**Ref D:** AC//AC ASC[22],

**Ref E:** $Cu(OH)_2$-Based Symmetric Supercapacitors[13].

**Table S7** GCD capacity of CoCN//CoCN ASSD with and without photoirradiation.

| Current density (mA cm$^{-2}$) | Discharge time without photoirradiation (s) | Discharge time with photoirradiation (s) | Capacity without photoirradiation (mF cm$^{-2}$) | Capacity with photoirradiation (mF cm$^{-2}$) | Current density (A g$^{-1}$) | Capacity without photoirradiation (F g$^{-1}$) | Capacity with photoirradiation (F g$^{-1}$) |
|---|---|---|---|---|---|---|---|
| 10.6 | 7.8 | 9.3 | 68.9 | 82.2 | 4.8 | 31.3 | 37.4 |
| 16.0 | 4.4 | 4.7 | 58.7 | 62.7 | 7.3 | 26.7 | 28.5 |
| 21.4 | 2.7 | 3.2 | 48.2 | 57.1 | 9.7 | 21.9 | 26.0 |
| 26.6 | 1.7 | 2.9 | 37.7 | 64.3 | 12.1 | 17.1 | 29.2 |

**Note:** The calculated capacity without photoirradiation is lower than that in **Figure 4c** because the electrolyte evaporated partially with photoirradiation.

**Table S8** The power density (P) and energy density (E) of CoCN//CoCN ASSD with and without photoirradiation.

| Energy density without photoirradiation (Wh kg$^{-1}$) | Power density without photoirradiation (Wh kg$^{-1}$) | Energy density with photoirradiation (Wh kg$^{-1}$) | Power density with photoirradiation (Wh kg$^{-1}$) |
|---|---|---|---|
| 13.8 | 6.4 | 16.4 | 6.4 |
| 11.7 | 9.6 | 12.5 | 9.6 |
| 9.6 | 12.9 | 11.4 | 12.8 |
| 7.5 | 16.0 | 12.9 | 16.0 |

**Table S9** Photocatalytic $H_2$ evolution amount under the simulated sunlight.

| Times / Samples | 0 h | 1h | 2h | 3h | 4h |
|---|---|---|---|---|---|
| **g-$C_3N_4$-1wt% Pt** | 0 | 2.08 | 17.44 | 39.44 | 73.42 |
| **g-$C_3N_4$** | 0 | 0.47 | 1.41 | 2.22 | 3.55 |
| **CoCN-0.14** | 0 | 0.68 | 2.04 | 5.31 | 7.60 |
| **CoCN-0.28** | 0 | 0.16 | 0.66 | 1.06 | 1.63 |
| **CoCN-0.55** | 0 | 1.41 | 2.82 | 4.34 | 5.57 |

Unit: μmol.

**Note:**

Photocatalytic results demonstrated that the doping amount of $Co_3O_4$ has a great influence on the photocatalytic $H_2$ evolution activity, and the optimal CoCN sample is CoCN-0.14. It may be rationalized that the introduction of $Co_3O_4$ can enhance the charge separation efficiency of g-$C_3N_4$ for promoting $H_2$ evolution, but excessive $Co_3O_4$ may act as the recombination centers and cover the active sites on the g-$C_3N_4$ surface, leading to the decreased photocatalytic activity. As a consequence, loading a suitable amount of $Co_3O_4$ is crucial for optimizing the photocatalytic performance.

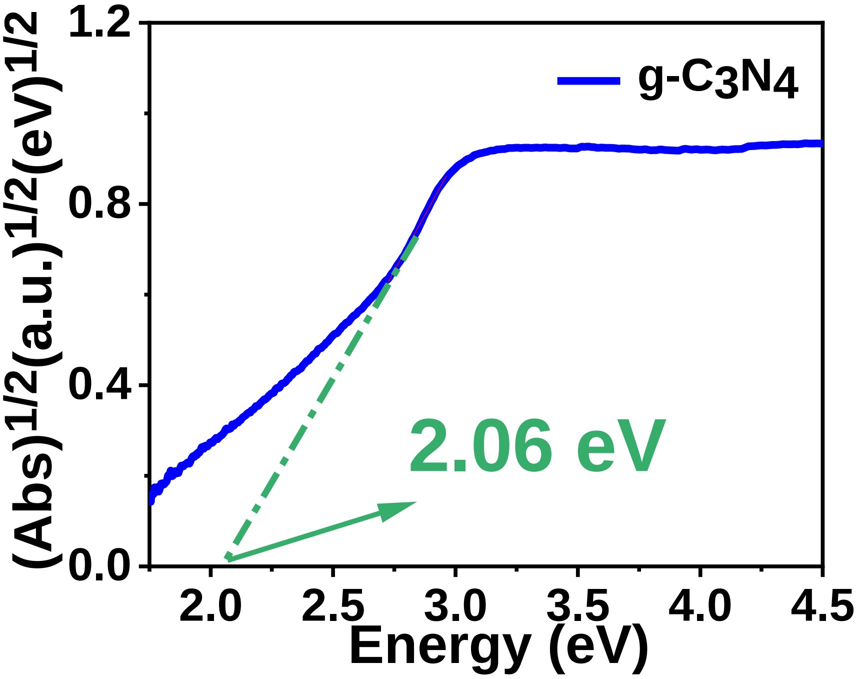


**Figure S31** Bandgap of g-$C_3N_4$.

**Note:**

The bandgap of the g-$C_3N_4$ is determined by optical absorption near the band edge by the following equation:

$$\alpha hv = A(hv - E_g)^{n/2} \quad \text{(S1)}$$

Where $hv$, $\alpha$, $A$, and $E_g$ are photonic energy, the optical absorption coefficient, the proportionality constant, and bandgap, respectively. The bandgap of the g-$C_3N_4$ is calculated to be 2.06 eV.

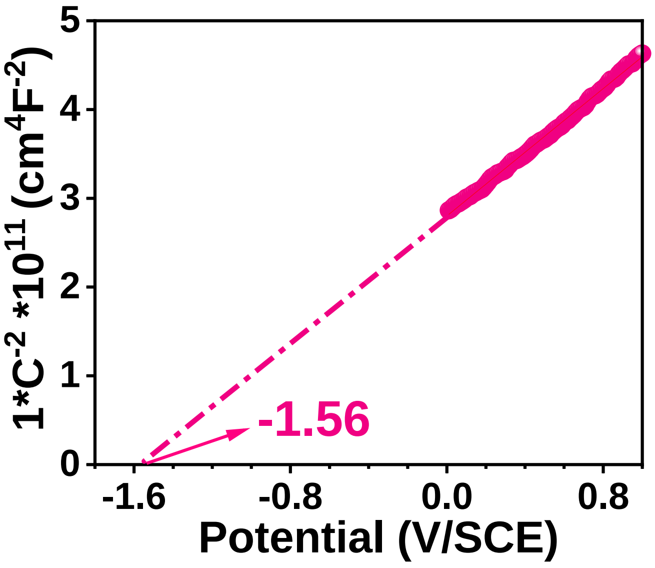


**Figure S32** Mott-Schottky curves of g-$C_3N_4$.

**Note:**

The tangent slope of the Mott-Schottky line is positive, indicating that the g-$C_3N_4$ has the characteristics of n-type semiconductor. The intercept is extrapolated and further processed in the Mott-Schottky formula:

$$\frac{1}{C^2} = \frac{2}{\varepsilon\varepsilon_0 N_D}\left(E - E_{fb} - \frac{K_B T}{q}\right) \tag{S2}$$

Where $\varepsilon$, $\varepsilon_0$, $C$, $N_D$, $T$, $E$, $q$, and $\kappa_B$ are the dielectric constants of free space, film electrode, space charge capacity, donor density, temperature, applied potential, electronic charge and the Boltzmann's constant, respectively.[5] Thus, the result is relative to the potential of the calomel electrode (flat band potential), which is converted to the standard hydrogen electrode (CB) of g-$C_3N_4$ as follows.

CB= -1.56 (flat band potential) + 0.2415 - (0.1~0.3) = -1.51 eV

**Note in Figure 5c:**

There are two space charge regions in the M-S curve of CoCN-0.14, that is, the n-type space charge region in the low voltage region and the p-type space charge region in the high voltage region. In the low voltage region, the slope of CoCN-0.14 is positive, which indicates that it has the characteristics of n-type g-$C_3N_4$ (Note in **Fig.S32**) and most of the carriers in the space charge region are electrons. when the potential is increased over ~0.30V, the slope of CoCN-0.14 becomes negative, the photoelectrode shows the characteristics of p-type semiconductor $Co_3O_4$[26,27], most of the carriers in the space charge region are holes[28].

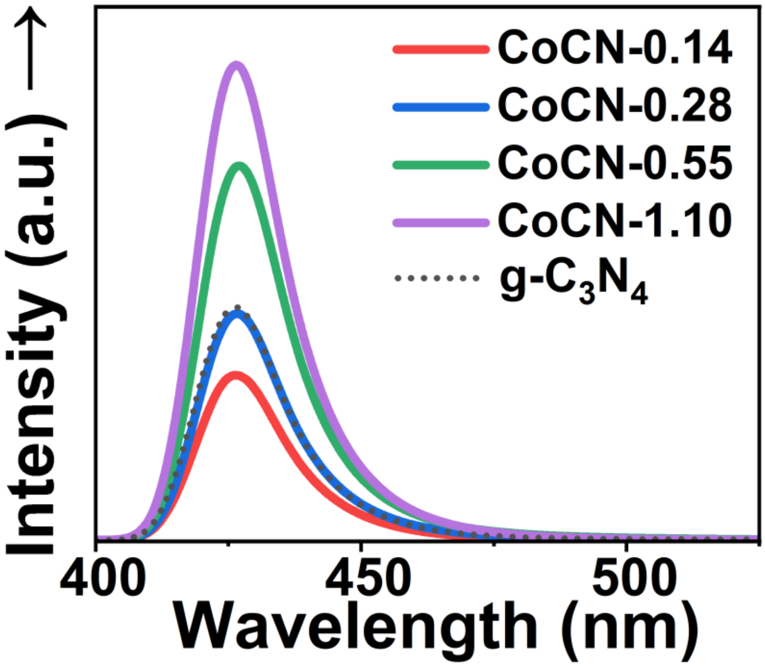


**Figure S33** Photoluminescence spectra of g-$C_3N_4$ and CoCN samples.

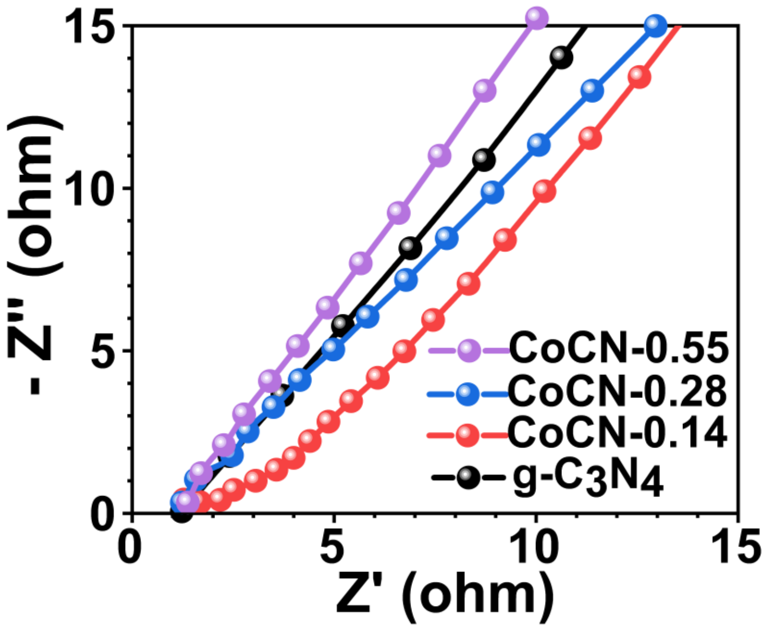


**Figure S34** Photoelectrochemical EIS of g-$C_3N_4$ and CoCN samples.

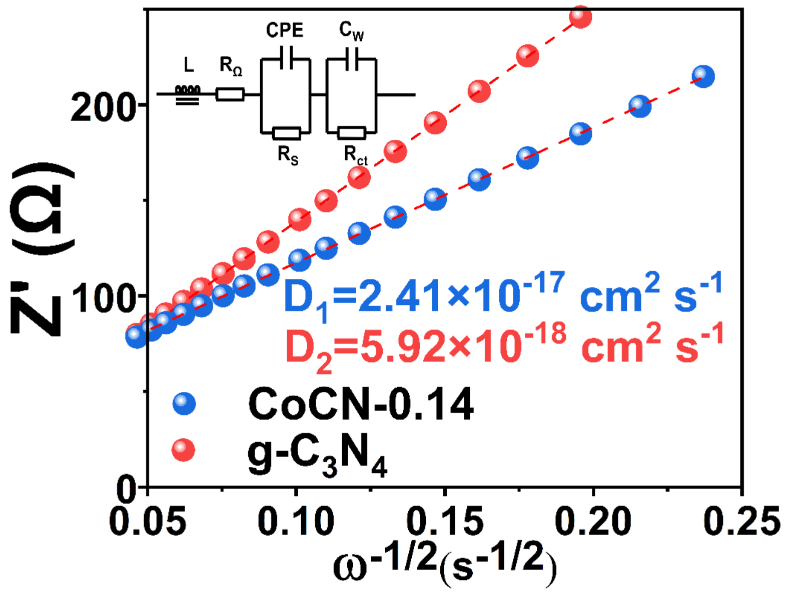


**Figure S35** $Z_{Re}$-$\omega^{-1/2}$ curve of the CoCN-0.14 and g-$C_3N_4$ ITO electrodes at a low-frequency region.

**Note:**

The Warburg coefficients ($\omega^{-1/2}$) of the CoCN-0.14 and g-$C_3N_4$ ITO electrode are 708.4 Ω $s^{-1/2}$ and 1110.0 Ω $s^{-1/2}$, respectively. The calculated $OH^-$ diffusion coefficient in 0.1M $Na_2SO_4$ aqueous system is $2.41\times10^{-17}$ $cm^2$ $s^{-1}$ and $5.92\times10^{-17}$ $cm^2$ $s^{-1}$, respectively, which shows that the migration rate of photogenerated carriers in the three-electrode system increases significantly by forming the $Co_3O_4$/ g-$C_3N_4$ p-n junction.

Combined with the photoluminescence results in **Figure S34**, the photogenerated carriers recombine to reduce the hydrogen production performance, and thus some electrons cannot be transferred to the electrode surface, increasing the interface charge transfer resistance ($\boldsymbol{R_{ct}}$). Finally, the $OH^-$ diffusion coefficient increases from $5.92\times10^{-18}$ $cm^2$ $s^{-1}$ to $2.41\times10^{-11}$ $cm^2$ $s^{-1}$ in **Figure S36**.

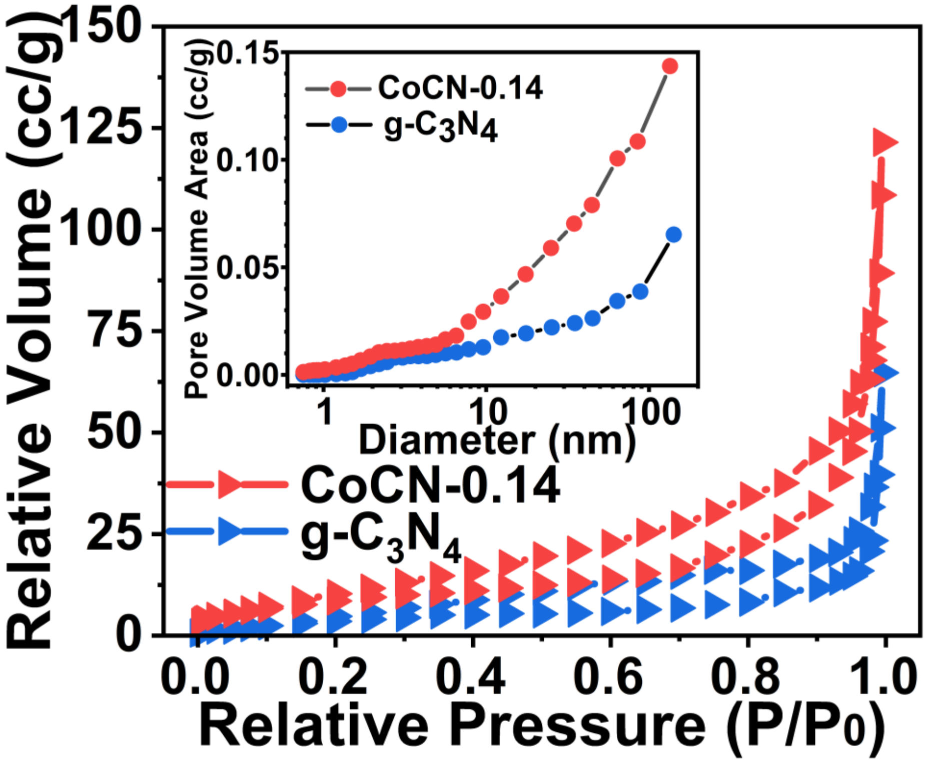


**Figure S36** The BET adsorption-desorption isotherms of g-$C_3N_4$ and CoCN-0.14.

**Note:**

The specific surface area of increases from 24.02 $m^2/g$ for g-$C_3N_4$ to 55.53 $m^2/g$ for CoCN-0.14, which is beneficial not only to the enriching of catalytic active sites but also to the improvement of capacity.

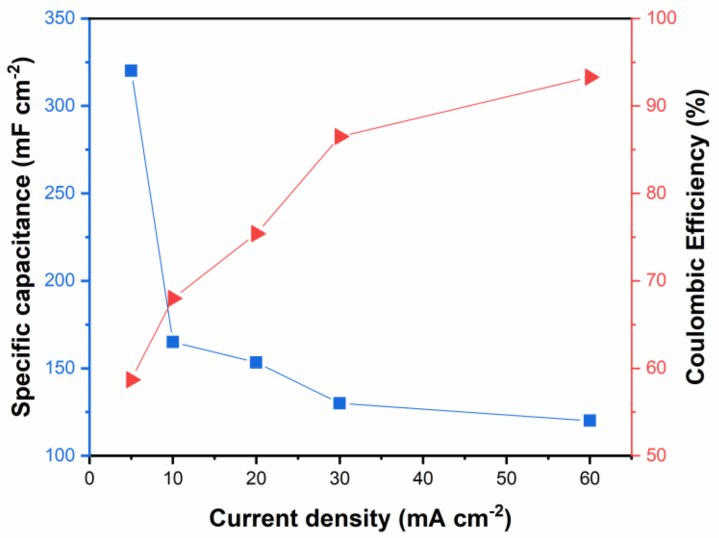


**Figure S37** Comparison of the Coulombic efficiencies of the CoCN//CoCN ASSD at different current densities.

**Notes:**

There were some side reactions happened in electrochemical reactions at a low current density, resulting in a non-symmetric charge/discharge curve in **Fig.4c**. With increasing current density, the charge/discharge time is shortened, and the electrochemical process is mainly restricted to the electric double layer, leading to enhanced Coulombic efficiency[29].

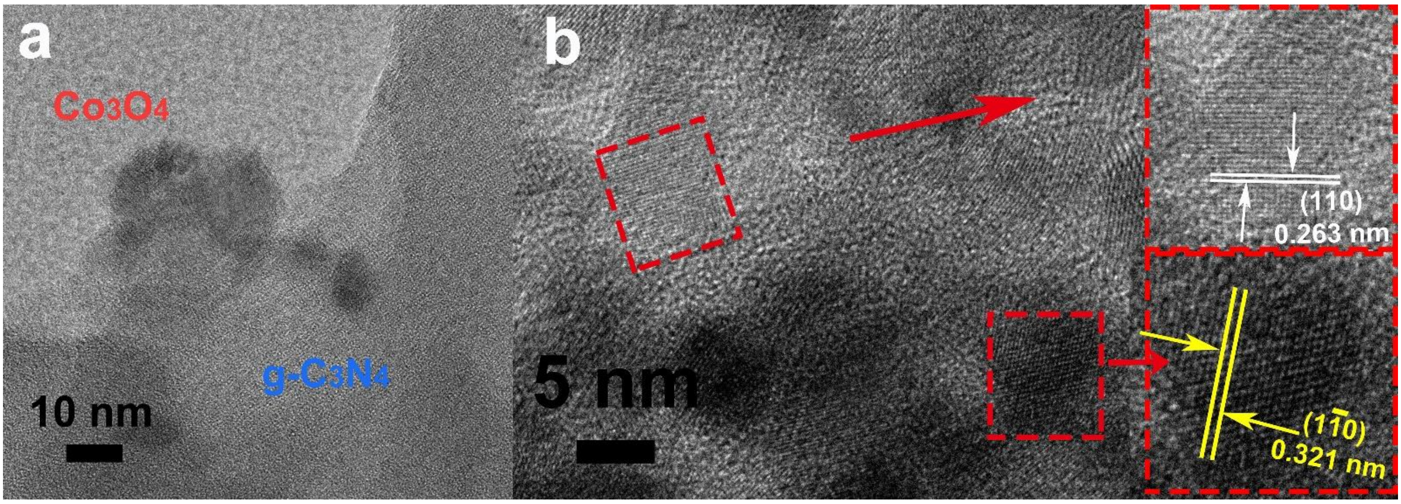


**Fig.S38** a) TEM and b) HRTEM image of CoCN-0.55 sample after photoirradiation.

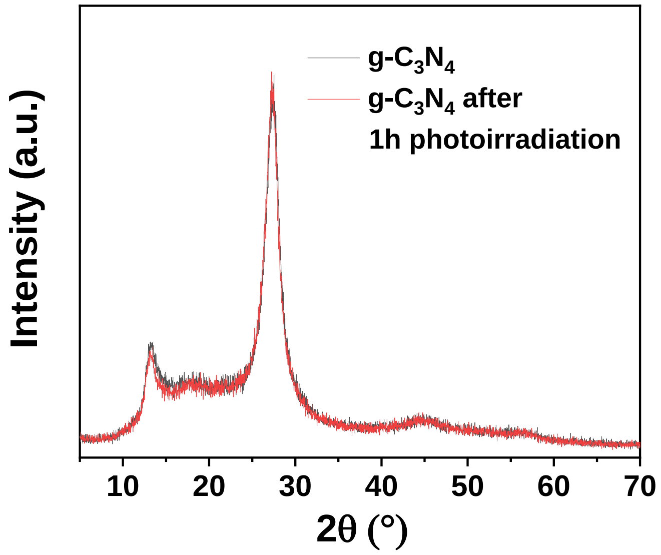


**Fig.S39** XRD patterns of g-$C_3N_4$ sample before and after photoirradiation for 1 h.

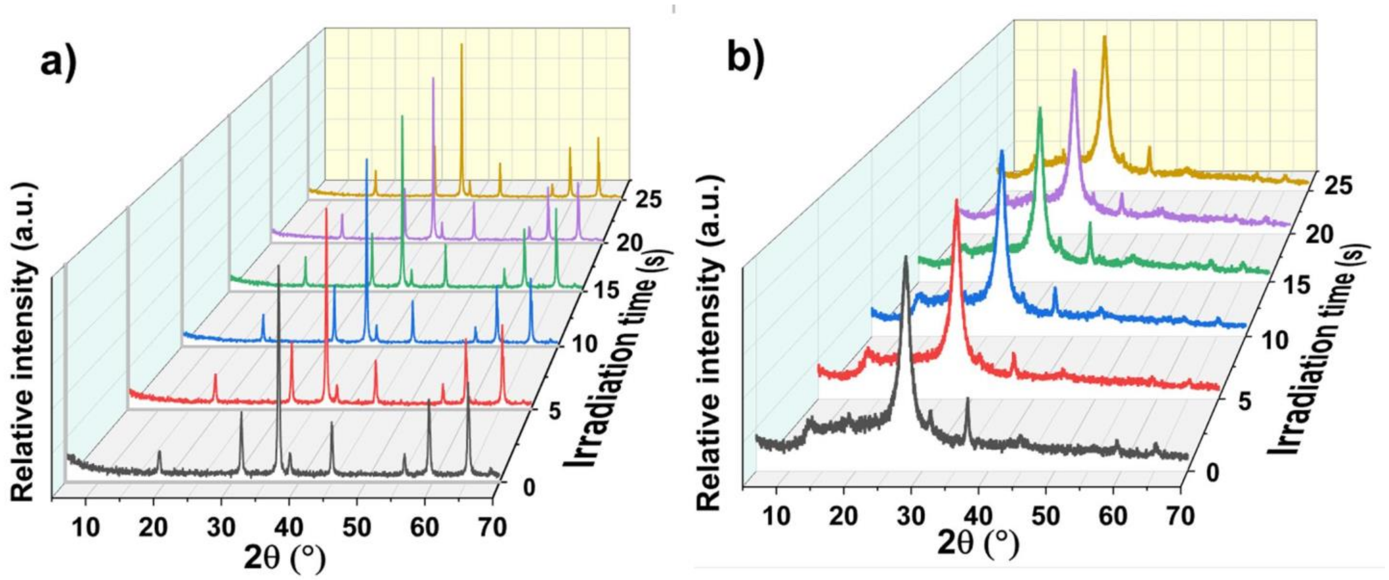


**Fig.S40** XRD patterns of a) $Co_3O_4$ and b) CoCN-0.55 samples after photoirradiation for 0-25s.

Note:

For the $Co_3O_4$ and CoCN-0.55 samples, there are no obvious changes in peak intensity and peak width after photoirradiation for 25 s. Slight variations in peak intensity and peak width are observed in the first 10 s, which, we believe, are related to phase, structural, or coordination changes under illumination.

## 4. References


[1] J. Li, Y. Wang, W. Xu, Y. Wang, B. Zhang, S. Luo, X. Zhou, C. Zhang, X. Gu, C. Hu, *Nano Energy* **2019**, *57*, 379.

[2] D. Zhang, Y. Zhang, X. Li, Y. Luo, H. Huang, J. Wang, P. K. Chu, *Journal of Materials Chemistry A* **2015**, *4*, 568.

[3] H. Liang, C. Xia, A. H. Emwas, D. H. Anjum, X. Miao, H. N. Alshareef, *Nano Energy* **2018**, *49*, 155.

[4] L. Bai, Y. Zhang, L. Zhang, Y. Zhang, L. Sun, N. Ji, X. Li, H. Si, *Nano Energy* **2018**, *53*, 982.

[5] S. Lin, Y. Zhang, Y. You, C. Zeng, X. Xiao, T. Ma, H. Huang, *Advanced Functional Materials* **2019**, *1903825*, 1903825.

[6] S. Gao, Z. Sun, W. Liu, X. Jiao, X. Zu, Q. Hu, Y. Sun, T. Yao, W. Zhang, S. Wei, Y. Xie, *Nature Communications* **2017**, *8*, 1.

[7] B. Ravel, M. Newville, *Journal of Synchrotron Radiation* **2005**, *12*, 537.

[8] P. Simon, Y. Gogotsi, *Nature Materials* **2008**, *7*, 845.

[9] L. Cao, G. Tang, J. Mei, H. Liu, *Journal of Power Sources* **2017**, *359*, 262.

[10] Y. Gao, W. Zhang, Q. Yue, B. Gao, Y. Sun, J. Kong, P. Zhao, *Journal of Power Sources* **2014**, *270*, 403.

[11] Y. W. Lee, B. S. Kim, J. Hong, H. Choi, H. S. Jang, B. Hou, S. Pak, J. Lee, S. H. Lee, S. M. Morris, D. Whang, J. P. Hong, H. S. Shin, S. N. Cha, J. I. Sohn, J. M. Kim, *Nano Energy* **2017**, *37*, 15.

[12] S. Shen, W. Guo, D. Xie, Y. Wang, S. Deng, Y. Zhong, X. Wang, X. Xia, J. Tu, *Journal of Materials Chemistry A* **2018**, *6*, 20195.

[13] P. Chang, H. Mei, Y. Zhao, W. Huang, S. Zhou, L. Cheng, *Advanced Functional Materials* **2019**, *1903588*, 1.

[14] J. Xu, N. Yang, S. Heuser, S. Yu, A. Schulte, H. Schönherr, X. Jiang, *Advanced Energy Materials* **2019**, *9*, 1.

[15] Y. Kang, Y. Yang, L.-C. Yin, X. Kang, L. Wang, G. Liu, H.-M. Cheng, *Advanced Materials* **2016**, *28*, 6471.

[16] J. Wang, Y. Shen, Y. Li, S. Liu, Y. Zhang, *Chemistry – A European Journal* **2016**, *22*, 12449.

[17] Y. Sun, S. Gao, F. Lei, J. Liu, L. Liang, Y. Xie, *Chemical Science* **2014**, *5*, 3976.

[18] X. Yin, C. Zhi, W. Sun, L.-P. Lv, Y. Wang, *Journal of Materials Chemistry A* **2019**, *7*, 7800.

[19] J. Hao, S. Peng, H. Li, S. Dang, T. Qin, Y. Wen, J. Huang, F. Ma, D. Gao, F. Li, G. Cao, *Journal of Materials Chemistry A* **2018**, *6*, 16094.

[20] S. Yang, Y. Liu, Y. Hao, X. Yang, W. A. Goddard, X. L. Zhang, B. Cao, *Advanced Science* **2018**, *5*, 1700659.

[21] Z. Shi, L. Xing, Y. Liu, Y. Gao, J. Liu, *Carbon* **2018**, *129*, 819.

[22] T. Liu, L. Zhang, W. You, J. Yu, *Small* **2018**, *14*, 1.

[23] E. K. Brooks, S. Der, M. T. Ehrensberger, *Materials Science and Engineering: C* **2016**, *60*, 427.

[24] Z. Li, S. Gadipelli, Y. Yang, G. He, J. Guo, J. Li, Y. Lu, C. A. Howard, D. J. L. Brett, I. P. Parkin, F. Li, Z. Guo, *Energy Storage Materials* **2019**, *17*, 12.

[25] C. Ji, F. Liu, L. Xu, S. Yang, *Journal of Materials Chemistry A* **2017**, *5*, 5568.

[26] X. Chang, T. Wang, P. Zhang, J. Zhang, A. Li, J. Gong, *Journal of the American Chemical Society* **2015**, *137*, 8356.

[27] P. Suyana, P. Ganguly, B. N. Nair, A. P. Mohamed, K. G. K. Warrier, U. S. Hareesh, *Environmental Science: Nano* **2017**, *4*, 212.

[28] F. Cardon, W. P. Gomes, *Journal of Physics D: Applied Physics* **1978**, *11*, L63.

[29] H. He, D. Sun, Y. Tang, H. Wang, M. Shao, *Energy Storage Materials* **2019**, *23*, 233.
